# PD-1 and TIM-3 differentially regulate subsets of mouse IL-17A–producing γδ T cells

**DOI:** 10.1084/jem.20211431

**Published:** 2022-12-07

**Authors:** Sarah C. Edwards, Ann Hedley, Wilma H.M. Hoevenaar, Robert Wiesheu, Teresa Glauner, Anna Kilbey, Robin Shaw, Katerina Boufea, Nizar Batada, Shinya Hatano, Yasunobu Yoshikai, Karen Blyth, Crispin Miller, Kristina Kirschner, Seth B. Coffelt

**Affiliations:** 1 Cancer Research UK Beatson Institute, Glasgow, UK; 2 School of Cancer Sciences, University of Glasgow, Glasgow UK; 3 Institute of Genetics and Molecular Medicine, University of Edinburgh, Edinburgh, UK; 4 Division of Immunology and Genome Biology, Medical Institute of Bioregulation, Kyushu University, Fukuoka, Japan; 5 Division of Host Defense, Medical Institute of Bioregulation, Kyushu University, Fukuoka, Japan

## Abstract

IL-17A–producing γδ T cells in mice consist primarily of Vγ6^+^ tissue-resident cells and Vγ4^+^ circulating cells. How these γδ T cell subsets are regulated during homeostasis and cancer remains poorly understood. Using single-cell RNA sequencing and flow cytommetry, we show that lung Vγ4^+^ and Vγ6^+^ cells from tumor-free and tumor-bearing mice express contrasting cell surface molecules as well as distinct co-inhibitory molecules, which function to suppress their expansion. Vγ6^+^ cells express constitutively high levels of PD-1, whereas Vγ4^+^ cells upregulate TIM-3 in response to tumor-derived IL-1β and IL-23. Inhibition of either PD-1 or TIM-3 in mammary tumor–bearing mice increased Vγ6^+^ and Vγ4^+^ cell numbers, respectively. We found that genetic deletion of γδ T cells elicits responsiveness to anti–PD-1 and anti–TIM-3 immunotherapy in a mammary tumor model that is refractory to T cell checkpoint inhibitors, indicating that IL-17A–producing γδ T cells instigate resistance to immunotherapy. Together, these data demonstrate how lung IL-17A–producing γδ T cell subsets are differentially controlled by PD-1 and TIM-3 in steady-state and cancer.

## Teaser

Coffelt, Edwards, and colleagues provide deep phenotypic analysis of mouse γδ T cells. They focus functional studies on IL-17A–producing γδ T cell subsets, showing that the co-inhibitory molecules, PD-1 and TIM-3, regulate cell expansion in tumor-bearing mice, which counteracts anti–PD-1 or anti–TIM-3 immunotherapy.

## Graphical Abstract

**Figure:**
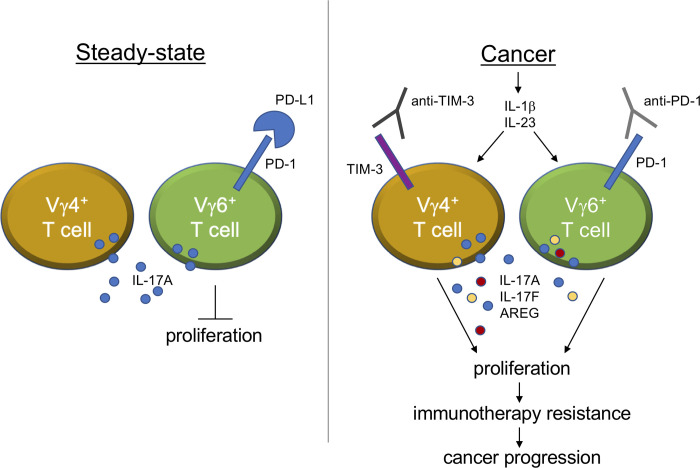

## Introduction

Mouse IL-17A–producing γδ T cells predominantly express Vγ4 or Vγ6 TCR chains (O’Brien and Born, 2020). In adult mice, Vγ4^+^ and Vγ6^+^ cells are self-renewing and persist for a long time in tissues (Sandrock et al., 2018). Functionally, these two subsets are indistinguishable. Their phenotype is almost identical, but there are some differences in the molecules they express, their behavior, and their distribution (O’Brien and Born, 2020). For example, the TCR repertoire of the Vγ6 population utilizes an invariant clone for the Vγ6Vδ1 pairing (Sandrock et al., 2018; Wei et al., 2015). The IL-17A–producing Vγ4^+^ cells comprise several semi-invariant oligoclonal TCRs that pair with δ4 or δ5 chains (Chen et al., 2019; Kashani et al., 2015; Wei et al., 2015). TCR expression levels are different between the two subsets with Vγ6^+^ cells expressing higher levels of CD3 subunits with their TCR (Paget et al., 2015). Vγ6^+^ cells are enriched in mucosal tissues such as the lung, dermis, and uterus as tissue resident cells (Monin et al., 2020; Sandrock et al., 2018; Tan et al., 2019); they are scarcely found in lymphoid organs of young mice, but accumulate in lymph nodes as mice age (Chen et al., 2019). Vγ4^+^ cells are more migratory than Vγ6^+^ cells, endowed with the ability to traffic from tissue to lymph nodes (McKenzie et al., 2017; Ramírez-Valle et al., 2015). In addition to IL-17A, these subsets can produce several other cytokines, such as IL-17F, IL-22, as well as the epidermal growth factor receptor ligand, amphiregulin (AREG; Jin et al., 2019; Sutton et al., 2009; Tan et al., 2019). Both Vγ4^+^ and Vγ6^+^ cells express the cell surface molecules CCR6, CCR2, CD44, IL-23R, IL-1R1, and IL-7Rα (Haas et al., 2009; McKenzie et al., 2017; Michel et al., 2012; Ribot et al., 2009; Sutton et al., 2009; Tan et al., 2019); the transcription factors RORγt and MAF (Zuberbuehler et al., 2019); as well as B lymphoid kinase (BLK; Laird et al., 2010). Vγ4^+^ cells express SCART2 and CD9, whereas Vγ6^+^ cells express SCART1 and PD-1 (Kisielow et al., 2008; Tan et al., 2019). During development, SOX4 and SOX13 transcription factors regulate the genesis of Vγ4^+^ cells (Gray et al., 2013; Malhotra et al., 2013), while promyelocytic leukemia zinc finger (PLZF) is required for Vγ6^+^ cells (Kreslavsky et al., 2009; Lu et al., 2015). Vγ6^+^ cells further differ from Vγ4^+^ cells in their unique regulation by β2 integrins and BCL-2 family members (McIntyre et al., 2020; Tan et al., 2019). Although several studies have shed light on the phenotypic similarities and differences between Vγ4^+^ and Vγ6^+^ cells in thymus, skin, and lymph nodes (Chen et al., 2019; Tan et al., 2019), how these subsets compare and how they are controlled in lung tissue is poorly understood.

Over the past few years, γδ T cells have garnered much attention in cancer, where unique subsets of γδ T cells can play either a pro-tumor or anti-tumor role (Lawrence et al., 2022; Silva-Santos et al., 2019). In mice, these functionally distinct γδ T cells can be stratified into two main subsets by the expression of the TNF receptor family member, CD27, which distinguishes IFNγ- from IL-17A–producing cells (Ribot et al., 2009). CD27^+^IFNγ^+^ γδ T cells that express Vγ1 or Vγ4 TCR chains have anti-tumor properties capable of direct cancer cell killing and boosting CD8^+^ T cell cytotoxic responses (Chen et al., 2019; Dadi et al., 2016; Gao et al., 2003; He et al., 2010; Lança et al., 2013; Liu et al., 2008; Lopes et al., 2021; Reis et al., 2022; Riond et al., 2009; Street et al., 2004). By contrast, CD27^−^IL-17A^+^ γδ T cells that express Vγ4 or Vγ6 TCR chains can drive primary tumor growth (Housseau et al., 2016; Jin et al., 2019; Ma et al., 2014; Patin et al., 2018; Rei et al., 2014; Reis et al., 2022; Rutkowski et al., 2015; Van Hede et al., 2017; Wakita et al., 2010), and they can promote metastasis (Baek et al., 2017; Benevides et al., 2015; Coffelt et al., 2015; Kulig et al., 2016; Wellenstein et al., 2019). One common mechanism that IL-17A–producing Vγ4^+^ and Vγ6^+^ cells share to foster cancer progression is the stimulation of granulopoiesis and recruitment of neutrophils to primary and secondary tumors that suppress anti-tumor CD8^+^ T cells. These immunosuppressive neutrophils are triggered by IL-17A–regulated G-CSF expression (Baek et al., 2017; Coffelt et al., 2015; Jin et al., 2019; Ma et al., 2014; Wellenstein et al., 2019). Within the lung microenvironment, γδ T cells are activated to produce IL-17A by tumor-derived or microbiota-induced IL-1β and IL-23 (Coffelt et al., 2015; Jin et al., 2019; Wellenstein et al., 2019), two cytokines with well-established influence on IL-17A–producing γδ T cells (Sutton et al., 2009). In addition, these pathways are highly comparable to human cancer. IL-17A–producing γδ T cells not only infiltrate human tumors (Boufea et al., 2020; Kargl et al., 2017; Wu et al., 2014), but high levels of IL-17A or abundance of γδ T cells also correlates with poor prognosis and metastasis in cancer patients (Benevides et al., 2015; Ma et al., 2012; Wu et al., 2014). Collectively, these studies underpin the crucial role of lung IL-17A–producing γδ T cells in cancer progression. However, questions remain about how these cells are locally controlled in the tumor-conditioned lung microenvironment.

Here, we performed a comprehensive analysis of γδ T cell phenotype, transcriptional diversity and function in healthy lung and mammary tumor–conditioned lung (i.e., the pre-metastatic niche) in mice. Using single-cell RNA sequencing (scRNAseq) and flow cytometry, we show that Vγ6^+^ cells in healthy lung are distinguishable from Vγ4^+^ cells by expression of a number of molecules, including CXCR6, JAML, NKG2D, and PD-1. Manipulation of PD-1 signaling on Vγ6^+^ cells altered intracellular signaling pathways as well as their proliferation. In a mouse model of breast cancer, scRNAseq revealed that the diversity of lung γδ T cells increases dramatically in response to a tumor. We found that both Vγ4^+^ and Vγ6^+^ cells proliferate, and Vγ4^+^ cells increase expression of IL-17A, IL-17F, and AREG, which is mediated by IL-1β and IL-23. While PD-1 expression remained at high levels on Vγ6^+^ cells in tumor-bearing mice, lung Vγ4^+^ cells up-regulated another co-inhibitory molecule, TIM-3. We demonstrate that inhibition of PD-1 or TIM-3 further increases Vγ6^+^ and Vγ4^+^ cell expansion, respectively. This expansion of Vγ6^+^ and Vγ4^+^ cells conferred resistance to anti–PD-1 or anti–TIM-3 immunotherapy, as the absence of these cells in tumor-bearing mice resulted in sensitivity to T cell checkpoint inhibitors. These data offer insight into the distinctive regulation of lung γδ T cell subsets in homeostasis and cancer.

## Results

### scRNAseq analysis identifies two clusters of γδ T cells in normal lung

**Figure S1.**
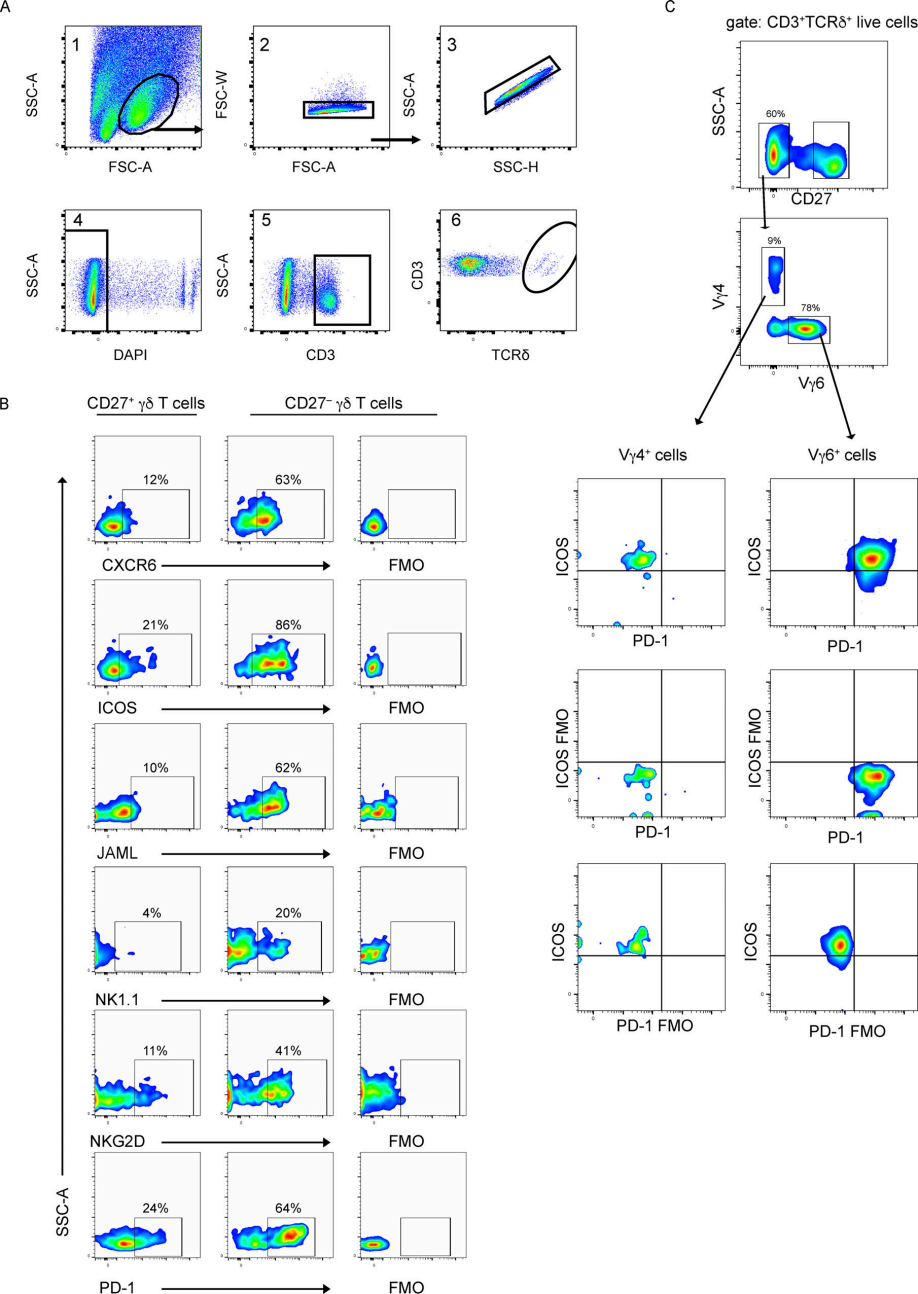
**Gating strategy for γδ T cell sorting and analysis. (A)** Single-cell suspensions of lung cells were stained with antibodies against CD3 and TCRδ. DAPI was used to exclude dead cells. Flow cytometry plots are depicted showing doublet exclusion, live cells, T cells, and γδ T cells (data related to Fig. 1; *n* = 4 WT mice). **(B)** Representative flow cytometry plots of indicated molecule expression in CD27^+^ and CD27^−^ γδ T cells from FVB/n mice. **(C)** Representative flow cytometry plots of indicated molecule expression in lung Vγ4^+^ and Vγ6^+^ cells from FVB/n mice. Numbers indicate percentage positive cells in gate. FMO = fluorescence minus one.

**Figure 1.**
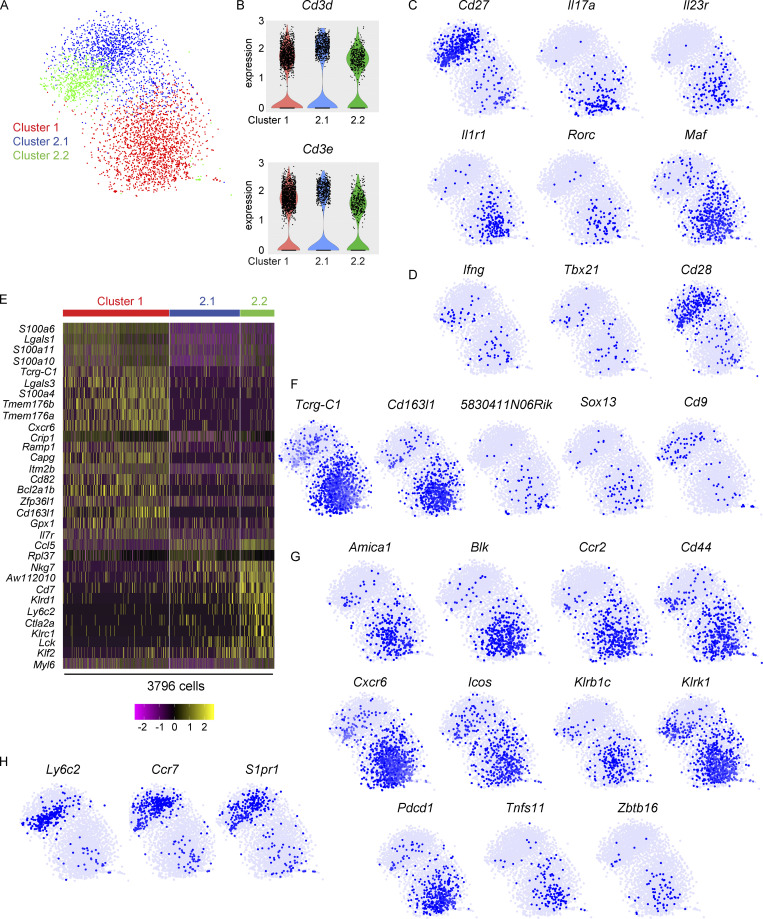
**scRNAseq identifies two major clusters of lung γδ T cells. (A)** Two-dimensional visualization of single γδ T cells from lungs of three WT FVB/n mice via tSNE. Each dot represents an individual cell (*n* = 3,796). **(B)** Violin plots of *Cd3d* and *Cd3e* expression for Clusters 1, 2.1, and 2.2. **(C and D)** Feature plots in tSNE map of indicated genes. **(E)** Heatmap showing *z*-score normalized expression of top 32 differentially expressed genes for Clusters 1, 2.1, and 2.2. Cells (*n* = 3,796) are plotted in columns by cluster, and genes are shown in rows. Gene expression is color coded with a scale based on z-score distribution, from −2 (purple) to 2 (yellow). **(F−H)** Feature plots in tSNE map of indicated genes.

To better understand γδ T cell heterogeneity in the lung, we performed scRNAseq of total CD3^+^TCRδ^+^ cells isolated from the lungs of WT FVB/n mice (Fig. S1 A). The isolated fraction comprised 2.5–5.3% of the viable cells. Libraries for scRNAseq were prepared using the Chromium 10× platform and we obtained single-cell transcriptomes from 3,796 γδ T cells from lungs of WT mice. Principal component analysis for dimensional reduction and unsupervised clustering were performed on the data, and t-distributed stochastic neighbor embedding (tSNE) was utilized for visualization of the data in two dimensions (Fig. 1 A). This unbiased transcriptional analysis of 3,796 individual cells segregated γδ T cells into two major clusters, which were designated as Cluster 1 and Cluster 2. Cluster 1 represented the largest number of γδ T cells within the lung compartment. Cluster 2 consisted of two transcriptionally related, yet distinct groups, which we labeled Clusters 2.1 and 2.2 (Fig. 1 A). Expression of *Cd3d* and *Cd3e* genes confirmed that all these cells are TCR-expressing cells (Fig. 1 B). To identify distinguishing characteristics separating Cluster 1 and Cluster 2, expression of a selected set of established marker genes often used to discriminate IL-17A–producing γδ T cells from IFNγ-producing γδ T cells was investigated within the scRNAseq dataset. *Cd27*, a co-stimulatory molecule, was chosen because its protein expression on γδ T cells stratifies IL-17A–producing (CD27^−^) cells from IFNγ-producing (CD27^+^) cells (Ribot et al., 2009). Expression of *Cd27* was largely absent from Cluster 1 and enriched in Clusters 2.1 and 2.2 (Fig. 1 C), suggesting that Cluster 1 may represent IL-17A–producing cells while Cluster 2 may represent IFNγ-producing cells. This hypothesis was confirmed when enrichment of *Il17a* and other genes associated with IL-17A signaling was observed in Cluster 1, such as the cytokine receptors *Il23r* and *Il1r1*, as well as the transcription factors *Rorc* and *Maf* (Fig. 1 C). Expression of *Ifng* and the gene encoding the transcription factor that regulates *Ifng* expression, *Tbx21* (T-Bet), was both localized to Cluster 2.2 and dispersed throughout Cluster 1 (Fig. 1 D). Expression of *Cd28*, which encodes a co-stimulatory molecule involved in IFNγ-producing γδ T cell expansion and IL-2 production (Ribot et al., 2012), was enriched in Cluster 2 (Fig. 1 D). These data indicate that the two major clusters of lung γδ T cells identified by scRNAseq are largely defined by *Cd27* expression and IL-17A signaling molecules in accordance with historical literature (Ciofani et al., 2012; Petermann et al., 2010; Ribot et al., 2009; Sutton et al., 2009; Zuberbuehler et al., 2019).

To uncover additional transcriptional differences between Clusters 1 and 2, differentially expressed genes defining each cluster were analyzed. The top 50 most significant genes from each cluster were combined to generate a heat map consisting of 32 genes (Fig. 1 E and Table S1). The clusters were defined by diverse expression profiles for TCR signaling, cytokines, cytokine receptors, chemokine receptors, co-stimulatory, and co-inhibitory molecules as well as natural killer (NK) cell markers. Cluster 1 representing CD27^−^ γδ T cells was enriched in genes from the S100 family of calcium-binding proteins, including *S100a4*, *S100a6*, *S100a10*, and *S100a11*. Members of the Galectin family including *Lgals1* (Galectin-1) and *Lgals3* (Galectin-3), which are carbohydrate binding proteins, were enriched in Cluster 1. Galectin-1–expressing γδ T cells are known to suppress antigen-specific anti-tumor immunity in a TLR5-dependent manner (Rutkowski et al., 2015). The ion channel homologues, *Tmem176a* and *Tmem176b*, were defining genes of Cluster 1. Expression of *Tmem176a* and *Tmem176b* is regulated by the *Il17a*-inducing transcription factor, RORγt (Ciofani et al., 2012). TMEM176A and TMEM176B have redundant functions in controlling ion influx/efflux in γδ T cells (Drujont et al., 2016). The chemokine receptor, *Cxcr6*, and the cytokine receptor, *Il7r*, appeared in the top 50 list of genes. Both receptors are established regulators of IL-17A–producing γδ T cell trafficking and cytokine expression (Butcher et al., 2016; Chen et al., 2019; Gray et al., 2011; Gray et al., 2012; Hayes et al., 1996; Michel et al., 2012). Cells in Cluster 1 expressed *Bcl2a1b* as well as *Bcl2a1a* and *Bcl2a1d* (Table S1), which are pro-survival factors that support Vγ6^+^ cells in skin (Tan et al., 2019). The other top genes for Cluster 1 included *Crip1*, *Ramp1*, *Capg*, *Itm2b*, *Cd82*, *Zfp36l1*, and *Gpx1* (Fig. 1 E). Vγ6^+^ cells make up the largest proportion of γδ T cells in the lung (Hayes et al., 1996; McIntyre et al., 2020). In agreement with these data, markers of Vγ6^+^ cells appeared in Cluster 1, which was the cluster containing the greatest number of cells (Fig. 1 A). These markers included *Tcrg-C1* and *Cd163l1*, which encodes the SCART1 protein (Fig. 1 F; Chen et al., 2019; Kisielow et al., 2008; Tan et al., 2019). The markers of Vγ4^+^ cells, including *5830411N06Rik* (the gene encoding SCART2), *Sox13*, and *Cd9* (Chen et al., 2019; Gray et al., 2013; Malhotra et al., 2013; Tan et al., 2019), were expressed by only a few cells in Cluster 1 (Fig. 1 F). Together, the defining genes of Cluster 1 identified for lung γδ T cells (e.g. *Tcrg-C1*, *Cd163l1*, *Cd27*, *Maf*, *S100a* genes, *Lgals1* and *Lgals1*, *Bcl2a1* genes, *Cxcr6*, etc.) are highly similar to the transcriptome of IL-17A–producing Vγ6^+^ cells from skin, thymus, adipose, uterus, and lymph nodes (Chen et al., 2019; Kohlgruber et al., 2018; Monin et al., 2020; Tan et al., 2019), suggesting that these commonalities can be used to identify Vγ6^+^ cells across various tissues.

Further investigation into the characteristics of Cluster 1 revealed additional insight into the transcriptome of these mostly Vγ6^+^ cells. Common genes for IL-17A–producing γδ T cells, such as *Blk*, *Cd44*, *Ccr2*, and *Zbtb16* (which encodes PLZF; Ciofani et al., 2012; Kohlgruber et al., 2018; Laird et al., 2010; McKenzie et al., 2017; Stark et al., 2005), were readily expressed in Cluster 1 cells (Fig. 1 G). Expression of the chemokine receptor, *Cxcr6*, and *Tnfsf11*, which encodes receptor activator of NF-κB ligand (RANKL), was more prevalent in Cluster 1 than Cluster 2. Two NK cell–associated molecules, *Klrb1c* and *Klrk1*, were highly enriched in Cluster 1. These genes encode NK1.1/CD161 and NKG2D, respectively, which are two proteins known for their involvement in target recognition and cancer cell killing. In addition, *Amica1* and *Icos*, two co-stimulatory receptors, and *Pdcd1* (PD-1), a co-inhibitory receptor, were enriched in Cluster 1 (Fig. 1 G). The *Amica1* gene encodes junction adhesion molecule-like (JAML), which is an activator of skin-resident, Vγ5 cells (Witherden et al., 2010). Inducible T cell costimulator (ICOS) signaling is important in thymic development of IL-17A–producing γδ T cells and function during experimental autoimmune encephalomyelitis (Buus et al., 2016; Galicia et al., 2009), while the function of PD-1 on these cells is unknown. Apart from the NK cell–associated genes, the Cluster 1 phenotype (e.g. *Amica1*, *Cxcr6*, *Icos*, *Pdcd1*, and *Tnfsf11*) was highly reminiscent of CD8^+^ tissue resident memory (T_rm_) T cells (Clarke et al., 2019; Djenidi et al., 2015; Kumar et al., 2017; Mackay et al., 2013; Peng et al., 2022; Strutt et al., 2018; Wein et al., 2019), indicating that Cluster 1 cells share many commonalities with antigen-experienced, non-circulating αβ T cells in the lung.

The defining genes of Cluster 2 represented TCR signaling molecules, cytotoxic molecules, cytokines, and T cell effector molecules, consistent with their CD27-expressing status and the established function of IFNγ-producing γδ T cells (Silva-Santos et al., 2019). Some of these genes included *Ccl5*, *Rpl37*, *Nkg7*, *AW112010*, *Cd7*, *Klrd1*, *Ly6c2*, *Ctla2a*, *Klrc1*, *Lck*, *Klf2*, and *Myl6* (Fig. 1 E). The product of the *Ly6c2* gene, Ly6C, is a molecule commonly associated with monocytes and neutrophils, and it can be expressed by CD27^+^ γδ T cells (Lombes et al., 2015), although its function on these cells is unknown. *Ly6c2* was expressed by Cluster 2.2 (Fig. 1 H). The KLF2 transcription factor is known to regulate γδ T cell trafficking through expression of sphingosine 1-phosphate receptor 1 (S1PR1; Odumade et al., 2010; Ugur et al., 2018). In addition to the top 50 genes (Fig. 1 E), the migratory-related molecules, *Ccr7* and *S1pr1*, were also a predominant feature of Cluster 2 with specific enrichment in Cluster 2.1 (Fig. 1 H). Taken together, these data uncover novel heterogeneity within the CD27^+^ compartment of γδ T cells.

### Lung Vγ6^+^ cells display a T_rm_ phenotype

**Figure 2.**
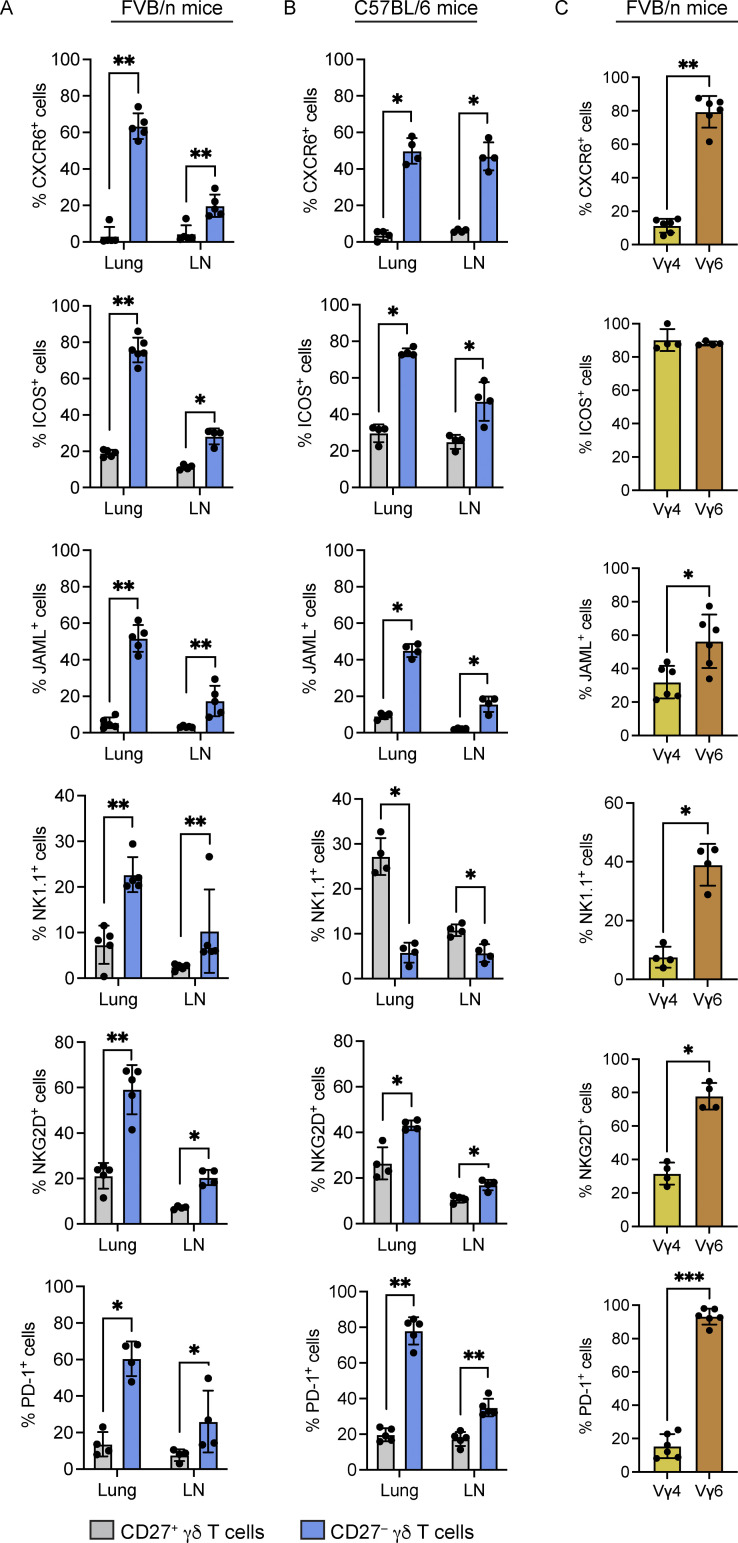
**Lung Vγ6**^**+**^ **cells express a T**_**rm**_ **phenotype.** Single-cell suspensions of lung and lymph nodes (LN) from FVB/n mice were stained with antibodies against CD3, TCRδ, CD27, and the indicated molecules. Cells were analyzed by flow cytometry. **(A and B)** Graphic representation of percentage positive cells for each indicated molecule in CD27^+^ and CD27^−^ γδ T cells from lung and LN of indicated mouse strain; each dot represents data from one mouse (*n* = 4–5 mice/group). Data are presented as mean ± SD. Mann–Whitney U test; *P < 0.05, **P < 0.01. **(C)** Expression of indicated molecules by lung Vγ4^+^ and Vγ6^+^ CD27^−^ cells. Each dot represents one mouse (*n* = 4–6 mice/group). Data are presented as mean ± SD. Mann–Whitney U test; *P < 0.05, **P < 0.01, and ***P < 0.001.

Having identified major transcriptional differences between lung γδ T cells by scRNAseq, we validated some of these differences at the protein level. We focused on the observation that cells from Cluster 1 expressed genes associated with T_rm_ cells (e.g. *Amica1*, *Cxcr6*, *Icos*, *Pdcd1*, and *Tnfsf11*) as well as the NK cell markers *Klrb1c* (NK1.1/CD161) and *Klrk1* (NKG2D). Flow cytometry analysis of lung γδ T cells isolated from FVB/n mice revealed that CD27^−^ γδ T cells expressed higher levels of CXCR6, ICOS, JAML, NK1.1, NKG2D, and PD-1, when compared to lung CD27^+^ γδ T cells (Fig. 2 A and Fig. S1 B), confirming the scRNAseq data and the similarity with CD8^+^ T_rm_ cells (Clarke et al., 2019; Djenidi et al., 2015; Kumar et al., 2017; Mackay et al., 2013; Peng et al., 2022; Strutt et al., 2018; Wein et al., 2019).

We also compared expression of these T_rm_ markers on CD27^−^ and CD27^+^ γδ T cells from the lymph node, which contains higher numbers of CD27^−^Vγ4^+^ cells and lower numbers of CD27^−^Vγ6^+^ cells than the lung. This analysis revealed that CXCR6, ICOS, JAML, NK1.1, NKG2D, and PD-1 are more highly expressed on CD27^−^ γδ T cells in a similar manner to cells from lung (Fig. 2 A). The reduced level of expression may reflect lower numbers of CD27^−^Vγ6^+^ cells in lymph nodes or tissue-specific influence over these cells.

NK1.1/CD161 is an established marker of IFNγ-producing CD27^+^ γδ T cells that distinguishes these cells from IL-17A–producing γδ T cells (Haas et al., 2009). Therefore, we hypothesized that the discrepancy between our data from lung and established literature may be explained by the differences in mouse strains. To this end, we analyzed lung and lymph node γδ T cells in C57BL/6J mice for the same proteins. CD27^−^ γδ T cells from C57BL/6J mice expressed higher levels of CXCR6, ICOS, JAML, NKG2D, and PD-1, when compared to CD27^+^ γδ T cells (Fig. 2 B), corroborating the observations in FVB/n mice. In contrast to FVB/n mice, however, NK1.1 expression was lower on CD27^−^ γδ T cells than CD27^+^ γδ T cells from C57BL/6J mice (Fig. 2 B). These data indicate that the T_rm_ phenotype of CD27^−^ γδ T cells is stable between mouse strains, while expression of NK1.1 (and likely other molecules) is dissimilar between strains.

We examined whether expression of CXCR6, ICOS, JAML, NK1.1, NKG2D, or PD-1 was specific to a particular γδ T cell subset. Flow cytometry analysis of lung CD27^−^ γδ T cells from WT FVB/n mice revealed that Vγ6^+^ cells expressed higher levels of all these molecules compared with Vγ4^+^ cells with the exception of ICOS, which was highly expressed by both populations (Fig. 2 C and Fig. S1 C). These data suggest that Vγ6^+^ cells and Vγ4^+^ cells may be governed by different molecules in homeostatic lung.

### PD-1 regulates expansion of lung Vγ6^+^ T cells

**Figure 3.**
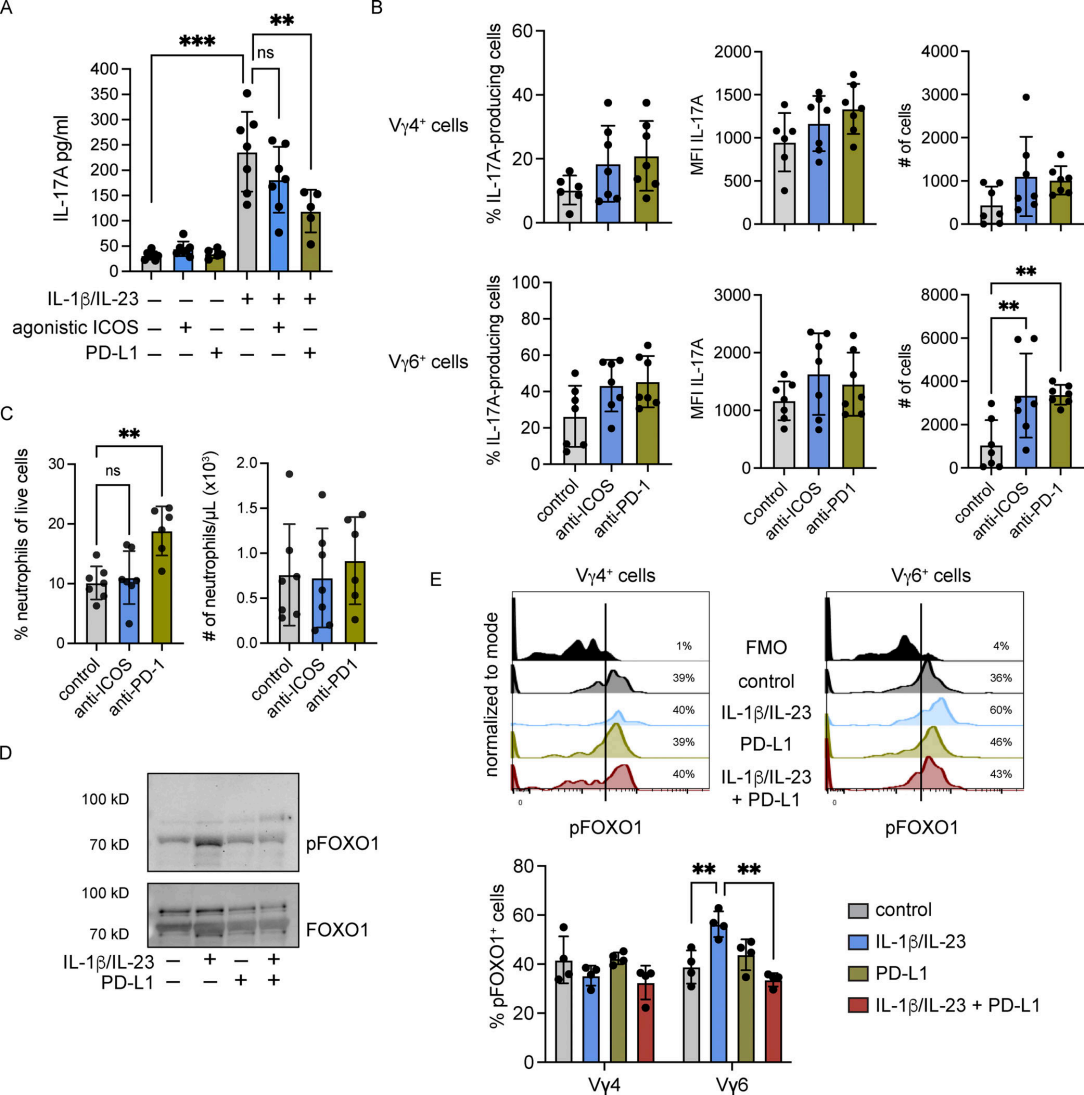
**PD-1 signaling regulates expansion of lung Vγ6**^**+**^ **cells. (A)** CD3^+^ T cells were isolated from the lungs of FVB/n mice and stimulated with recombinant IL-1β and IL-23 in the presence of plate-bound PD-L1-Fc or plate-bound anti-ICOS for 24 h. Supernatants were examined for IL-17A levels by ELISA. Each dot represents cells from one mouse (*n* = 5–7/group). Data are presented as mean ± SD. One-way ANOVA followed by Tukey’s posthoc test; **P < 0.01, ***P < 0.001. **(B)** WT FVB/n mice were injected with a single dose of 200 μg anti–PD-1 or anti-ICOS followed by injections of 100 μg for two consecutive days. Control mice followed the same dosage regime with isotype control. Mice were sacrificed 24 h after the third injection. Single-cell suspensions from lung were stimulated for 3 h with PMA, ionomycin, and Brefeldin A. Cells were stained with antibodies against CD3, TCRδ, CD27, Vγ4, Vγ6, and IL-17A. The proportion of cells expressing IL-17A, the MFI of IL-17A, and the absolute number of cells is represented graphically for both lung Vγ4^+^ and Vγ6^+^ CD27^−^ cells. Each dot represents one mouse. Data are presented as mean ± SD (*n* = 6–7 mice/group). One-way ANOVA followed by Dunnett’s posthoc test; **P < 0.01. **(C)** Percentage and total numbers of neutrophils in isotype control, anti–PD-1- and anti-ICOS–treated mice as measured by IDEXX ProCyte hematology analyzer. Each dot represents one mouse. Data are presented as mean ± SD (*n* = 6–7 mice/group). One-way ANOVA followed by Dunnett’s posthoc test; **P < 0.01. **(D)** Representative Western blot analysis (from three biological replicates) of phospho-FOXO1 and total FOXO1 levels in γδ T cells from lungs of FVB/n mice stimulated as depicted. **(E)** CD3^+^ T cells were isolated from the lungs of FVB/n mice and cultured with plate-bound PD-L1-Fc for 3 h. Cells were stimulated with recombinant IL-1β and IL-23 for the last 30 min. Cells were analyzed by flow cytometry. Representative histograms are shown, and combined data are represented graphically. Each dot represents one mouse. Data are presented as mean ± SD (*n* = 4 mice/group). One-way ANOVA followed by Dunnett’s posthoc test; **P < 0.01.

We were intrigued by the high expression of ICOS and PD-1 on lung Vγ6^+^ cells. PD-1, through its interaction with PD-L1 or PD-L2, functions as a negative regulator of T cell activation by interfering with TCR signaling (Honda et al., 2014). ICOS is a co-stimulatory molecule primarily expressed by CD4^+^ T helper and regulatory cells (Amatore et al., 2020), but it is also important for development and regulation of IL-17A–producing γδ T cells (Buus et al., 2016; Galicia et al., 2009). Therefore, we hypothesized that ICOS and PD-1 are negative regulators of Vγ6^+^ cells. To address this hypothesis, we stimulated lung CD3^+^ T cells with IL-1β and IL-23 in the presence of recombinant PD-L1 or an ICOS agonistic antibody and measured IL-17A by ELISA. IL-17A levels in conditioned medium were increased by IL-1β and IL-23 stimulation. However, the addition of PD-L1 co-stimulation diminished IL-1β/IL-23–induced IL-17A production, while ICOS co-stimulation failed to significantly affect IL-17A levels (Fig. 3 A). These data indicate that activation of the PD-1 pathway functions to limit IL-17A expression in lung γδ T cells. Consequently, we examined the effect of anti–PD-1 and anti-ICOS blocking antibodies on lung γδ T cells in vivo. Naive FVB/n mice were treated for three consecutive days with anti–PD-1, anti-ICOS, or isotype control antibody; consequently, IL-17A expression by lung γδ T cells was measured by intracellular flow cytometry. Contrary to the ELISA data, expression of IL-17A—either the proportion or median fluorescence intensity (MFI)—in Vγ4^+^ or Vγ6^+^ cells remained unchanged by inhibition of ICOS or PD-1 signaling (Fig. 3 B). We calculated the absolute number of Vγ4^+^ or Vγ6^+^ cells to determine whether anti-ICOS or anti–PD-1 had any effect on cell expansion. While the number of Vγ4^+^ cells remained the same, Vγ6^+^ cells in the lungs increased in mice treated with anti-ICOS or anti–PD-1 (Fig. 3 B), indicating that these molecules control proliferation or survival of Vγ6^+^ cells. We hypothesized that the increase in total Vγ6^+^ cells by anti-ICOS and anti–PD-1 results in a systemic increase in IL-17A. Since IL-17A is an essential cytokine for neutrophil expansion (Coffelt et al., 2015), we measured neutrophils in the blood of isotype control–, anti–PD-1- and anti-ICOS–treated WT mice. We found that the frequency of blood neutrophils is increased by anti–PD-1 treatment but anti-ICOS treatment failed to affect neutrophils when compared to controls (Fig. 3 C). The total number of neutrophils in blood remained the same between groups (Fig. 3 C). Taken together, these data indicate that PD-1 is a negative regulator of lung Vγ6^+^ cells, whose activation limits expansion of these cells, while the role of ICOS signaling on these cells remains unclear.

**Figure S2.**
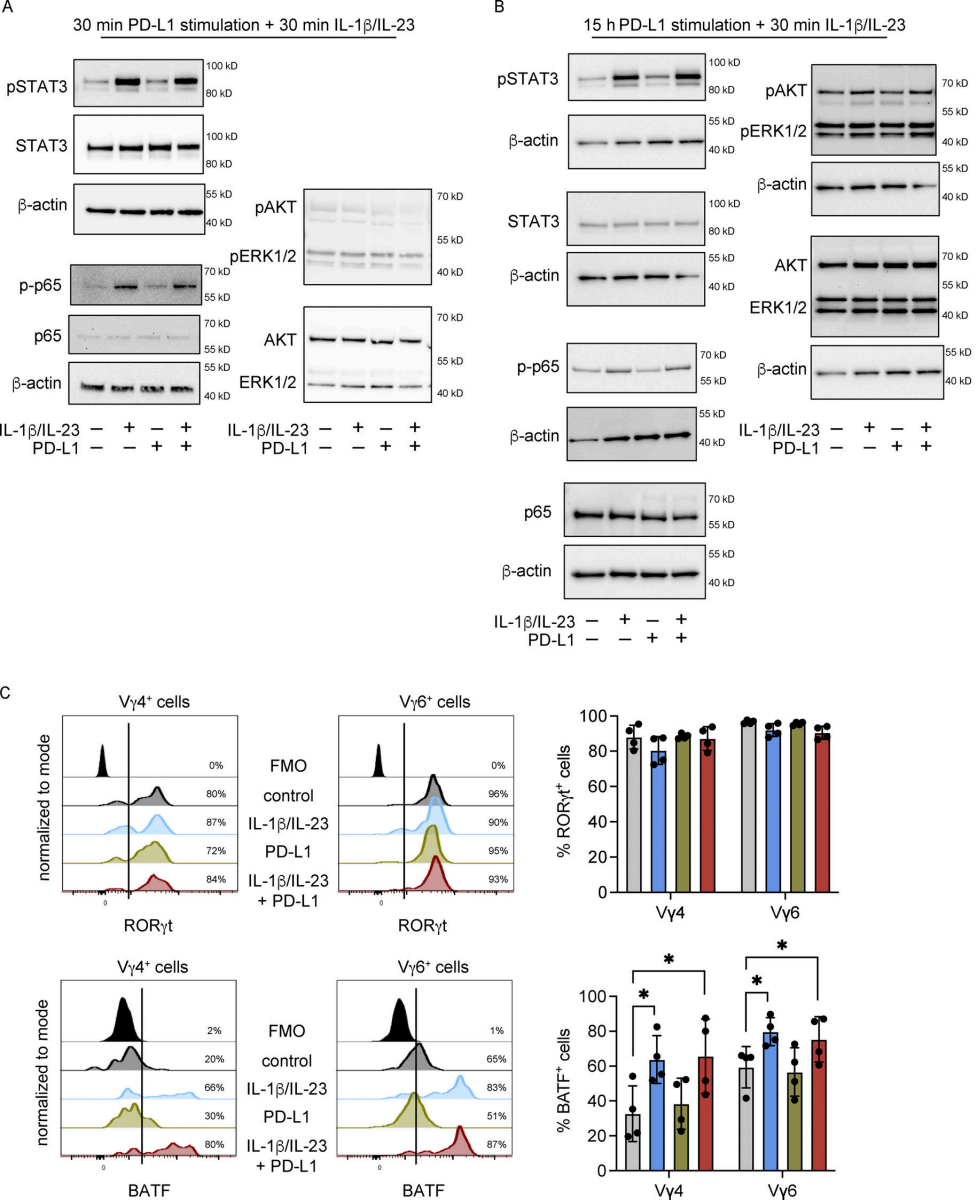
**PD-1 activation on γδ T cells fails to modulate JAK/STAT and NF-κB pathways induced by IL-1β and IL-23. (A)** γδ T cells were isolated from lungs of WT FVB/n mice and stimulated as indicated. Phosphorylated proteins and total protein levels were analyzed by Western blot. Representative images are shown from three biological replicates. The β-actin blot under the STAT3 blots is a loading control for the pSTAT3 and total STAT3 blots and a sample integrity control for AKT/ERK blots. The β-actin loading control is shown for p65 and p-p65. **(B)** γδ T cells were isolated from lungs of *Tcrb*^*−/−*^ mice on C57Bl/6 background and stimulated as indicated. Phosphorylated proteins and total protein levels were analyzed by Western blot. Representative images are shown from three to five biological replicates. For STAT3 and AKT/ERK analysis, one blot was probed for phosphorylated STAT3, stripped, and reprobed for total AKT/ERK, while another blot containing the same samples was probed for phospho-AKT/ERK then total STAT3. The β-actin blots under each panel correspond to loading controls for the above panel so these are duplicated for STAT3 and AKT/ERK blots. β-actin loading control blots are shown for the p65 and p-p65 blots. **(C)** CD3^+^ T cells were isolated from the lungs of FVB/n mice and cultured with plate-bound PD-L1-Fc for 3 h. Cells were stimulated with recombinant IL-1β and IL-23 for the last 30 min. Cells were analyzed by flow cytometry. Representative histograms are shown, and combined data are shown graphically for total RORγt and BATF levels. Each dot represents cells from one mouse. Data are presented as mean ± SD (*n* = 4 mice/group). One-way ANOVA followed by Dunnett’s posthoc test; *P < 0.05.

To further investigate how PD-1 stimulation impacts Vγ6^+^ cells, we measured intracellular signaling pathways after IL-1β/IL-23 treatment with or without PD-L1 engagement. As IL-1β and IL-23 activate NK-κB and STAT3 pathways, respectively, we analyzed phosphorylation of these proteins by Western blot. IL-1β/IL-23 treatment of total lung γδ T cells increased phosphorylation of p65 and STAT3; however, short-term or long-term PD-L1 engagement failed to affect this activation (Fig. S2, A and B). In addition, IL-1β/IL-23 treatment and PD-L1 engagement on γδ T cells failed to induce PI3K/AKT (PKB, protein kinase B) and MAPK pathways (Fig. S2, A and B). We then tested whether the expression of RORγt and basic leucine zipper ATF-like transcription factor (BATF), two transcription factors that control *Il17a* transcription, was influenced by PD-L1 engagement. PD-1 signaling is known to activate the transcription factor BATF to impair CD8^+^ T cell proliferation (Quigley et al., 2010), and BATF-deficient IL-17A–producing γδ T cells are hyper-proliferative (Barros-Martins et al., 2016; McKenzie et al., 2017). This flow cytometric analysis showed that RORγt is expressed at high levels in Vγ4^+^ and Vγ6^+^ cells, but is not affected by IL-1β/IL-23 or PD-L1 stimulation (Fig. S2 C). BATF expression was increased by cytokine treatment in both Vγ4^+^ and Vγ6^+^ cells, and this upregulation remained unchanged by PD-L1 engagement (Fig. S2 C); therefore, we ruled out a role for PD-1 signaling in RORγt or BATF inhibition. We also investigated phosphorylation of FOXO1 by Western blot, since this transcription factor inhibits RORγt (Lainé et al., 2015) and functions downstream of TCR and PD-1 signaling in αβ T cells (Staron et al., 2014). IL-1β/IL-23 treatment of lung γδ T cells resulted in phosphorylation of FOXO1 (Fig. 3 D). The phosphorylation of FOXO1 by the cytokines was reduced back to baseline when γδ T cells were cultured with PD-L1 (Fig. 3 D). We then used flow cytometry to determine whether FOXO1 phosphorylation is modulated the same or differently in Vγ4^+^ and Vγ6^+^ cells. This experiment showed that IL-1β/IL-23 stimulates FOXO1 phosphorylation specifically in Vγ6^+^ cells without modulating FOXO1 in Vγ4^+^ cells. Moreover, incubation of Vγ6^+^ cells (but not Vγ4^+^ cells) with PD-L1 reduced phosphorylation of FOXO1 (Fig. 3 E). These results suggest that PD-1 signaling functions as a negative regulator of Vγ6^+^ cells through modulation FOXO1 activity.

### The T_rm_ phenotype of Vγ6^+^ cells is largely unaffected by tumor-derived factors

**Figure 4.**
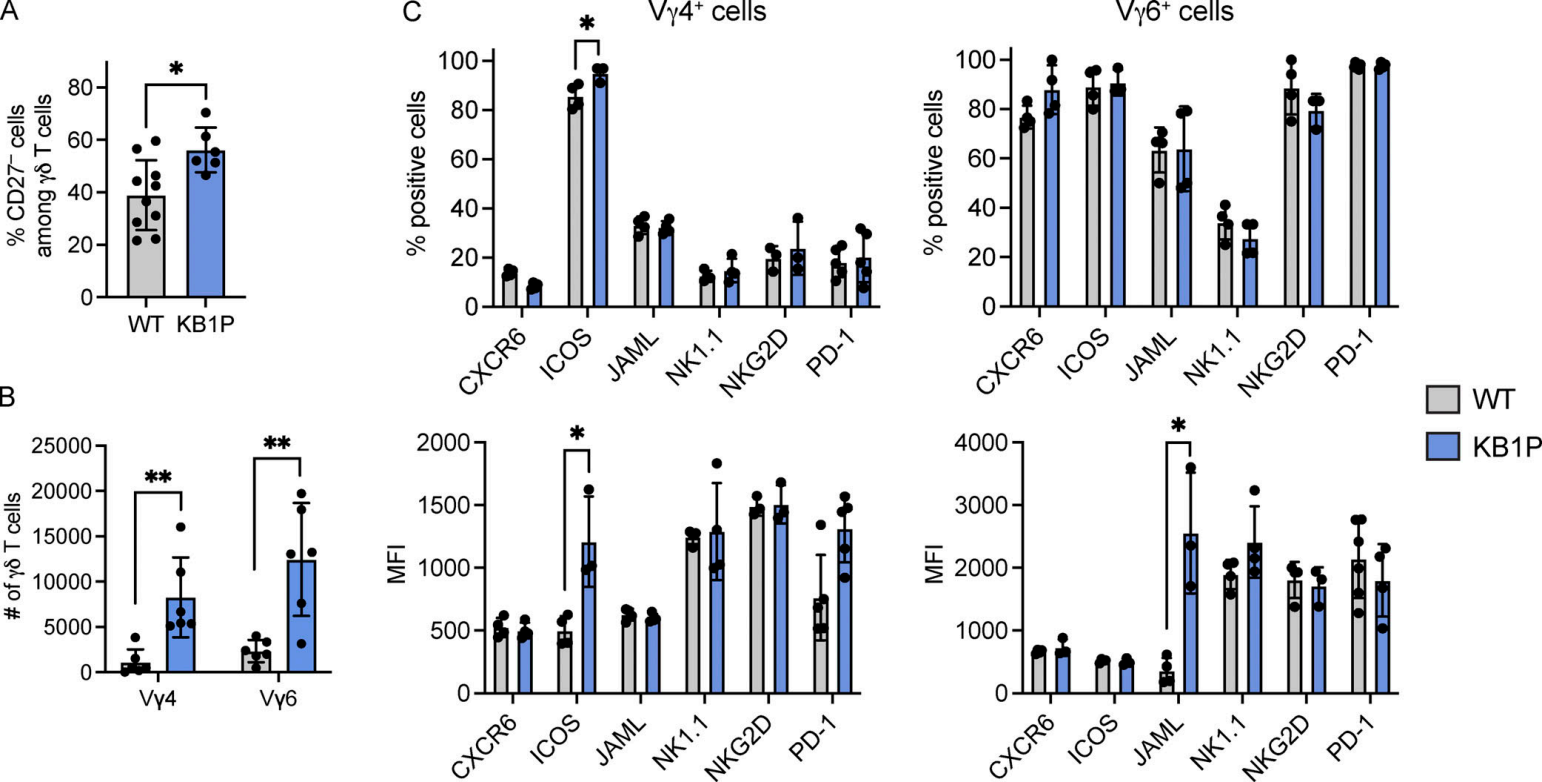
**Tumors in the mammary gland drive lung γδ T cell expansion. (A)** Frequency of CD27^−^ γδ T cells in WT and tumor-bearing *K14-Cre*;*Brca1*^*F/F*^;*Trp53*^*F/F*^ (KB1P) mice (*n* = 10 WT, 6 KB1P) as determined by flow cytometry. Each dot represents one mouse. Data are presented as mean ± SD. Mann–Whitney U test; *P < 0.05. **(B)** Absolute number of Vγ4^+^ and Vγ6^+^ CD27^−^ cells in the lungs of WT and tumor-bearing KB1P mice (*n* = 6 WT, 6 KB1P) as determined by flow cytometry. Each dot represents one mouse. Data are presented as mean ± SD. Mann–Whitney U test; **P < 0.01. **(C)** Expression and MFI of CXCR6, ICOS, JAML, NK1.1, NKG2D, and PD-1 by lung CD27^−^ γδ T cell subsets from WT and tumor-bearing KB1P mice (*n* = 3–5 WT, KB1P). Each dot represents one mouse. Data are presented as mean ± SD. Mann-Whitney U test; *P < 0.05.

Since lung Vγ4^+^ and Vγ6^+^ cells that produce IL-17A are important mediators of cancer progression and metastasis (Coffelt et al., 2015; Jin et al., 2019; Kulig et al., 2016), we examined the impact of tumor conditioning on these subsets. We used a mouse model of breast cancer in which the γδ T cell–IL-17A–neutrophil axis is established (Coffelt et al., 2015; Wellenstein et al., 2019): the *K14-Cre*;*Brca1*^*F/F*^;*Trp53*^*F/F*^ (KB1P) model of triple negative breast cancer, which develops invasive ductal carcinoma in one or more mammary glands at around 8 mo of age (Liu et al., 2007). When comparing tumor-bearing KB1P mice with tumor-free WT mice, the proportion of CD27^−^ γδ T cells and the total number of Vγ4^+^ and Vγ6^+^ cells was higher in the lungs of tumor-bearing mice (Fig. 4, A and B). These data indicate that tumors in the mammary gland drive γδ T cell expansion in the pre-metastatic lung. Phenotypic analysis of the markers we identified by scRNAseq revealed that expression of CXCR6, ICOS, JAML, NK1.1, NKG2D, and PD-1 by Vγ4^+^ and Vγ6^+^ cells remains unaffected by mammary tumor conditioning (Fig. 4 C). There were two exceptions to this observation: the proportion and MFI of ICOS on Vγ4^+^ cells as well as MFI of JAML on Vγ6^+^ cells was increased in tumor-bearing KB1P mice (Fig. 4 C). These data suggest that the T_rm_ phenotypic markers identified for lung Vγ6^+^ cells are largely constant between WT and mammary tumor–bearing mice, while the phenotype of Vγ4^+^ cells is somewhat more responsive to tumors.

### Lung γδ T cell diversity increases in response to mammary tumors

**Figure 5.**
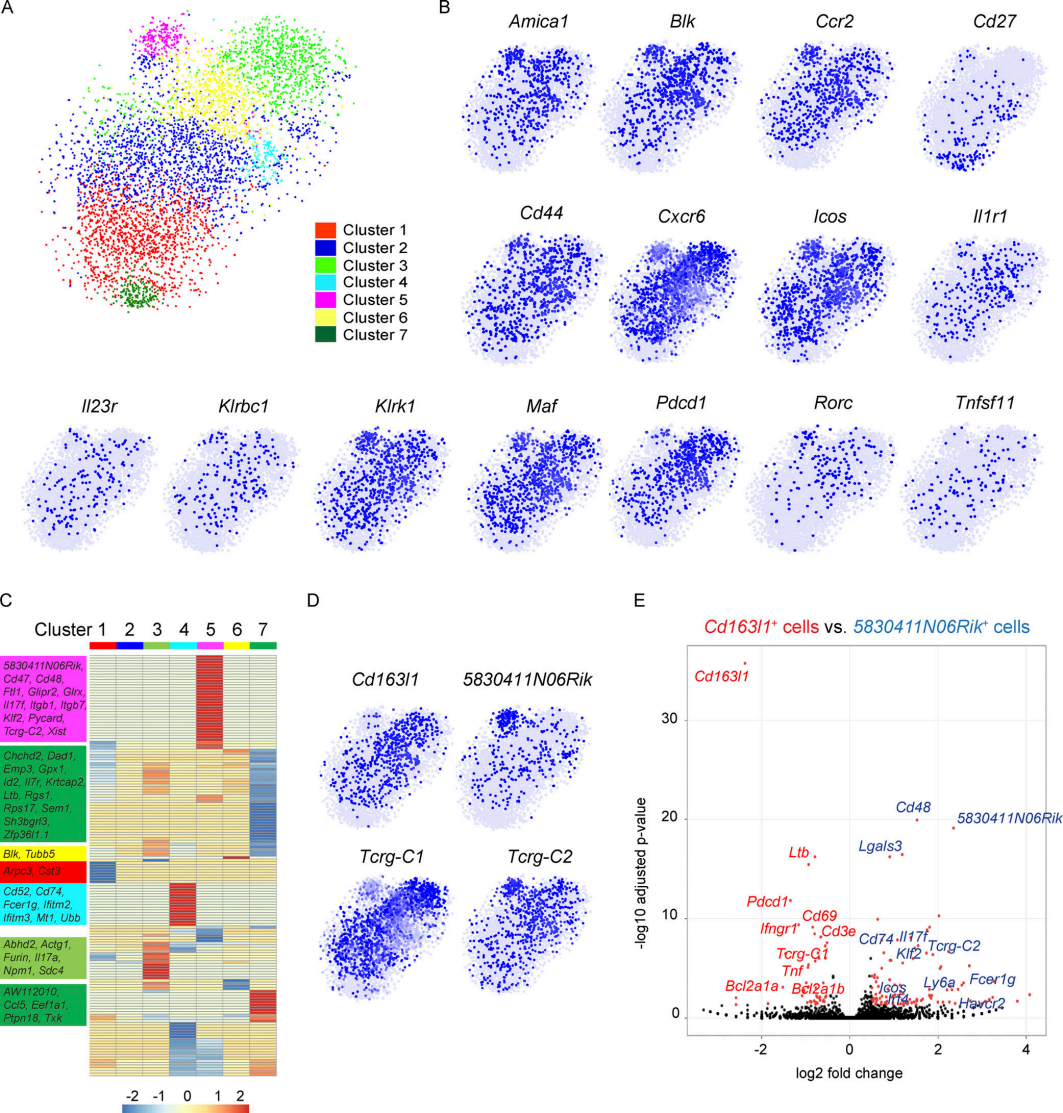
**Mammary tumors instigate increased heterogeneity of lung γδ T cells. (A)** Two-dimensional visualization of single γδ T cells from lungs of three tumor-bearing KB1P mice via tSNE. **(B)** Feature plots in tSNE map of indicated genes. **(C)** Heatmap showing z-score normalized expression of 278 differentially expressed genes for Clusters 1–7 (from Fig. 4 C). Clusters are plotted in columns, and genes are shown in rows. Gene expression is color coded with a scale based on z-score distribution, from −2 (blue) to 2 (red). Selected genes specific to each cluster that do not overlap with another cluster are shown on left side in boxes color coded by cluster. Full gene lists are provided in Table S2. **(D)** Feature plots in tSNE map of indicated genes. **(E)** The transcriptomes of *Cd163l1*- and *5830411N06Rik*-expressing cells from A were compared with each other and represented as a volcano plot. Genes highly expressed by *Cd163l1*-expressing cells are denoted in red, and genes highly expressed by *5830411N06Rik*-expressing cells are denoted in blue.

To gain a deeper understanding of lung Vγ4^+^ and Vγ6^+^ cell phenotype in KB1P tumor-bearing mice, we performed scRNAseq analysis on total lung γδ T cells from KB1P mice. From the computational interrogation of 5,091 individual cells, clustering analysis revealed that lung γδ T cells segregate into seven unique clusters (Fig. 5 A), which was remarkably different from the two clusters observed in WT mice (Fig. 1 A). Clusters 1–6 mostly lacked expression of *Cd27* mRNA, whereas Cluster 7 was enriched in cells expressing *Cd27* (Fig. 5 B), indicating that lung CD27^−^ γδ T cells are highly responsive to mammary tumors. This observation reflected the expansion of CD27^−^ γδ T cells we found by flow cytometry (Fig. 4, A and B). We evaluated the data for expression of genes identified from WT scRNAseq analysis and other common marker genes for IL-17A–producing γδ T cells, including *Amica1*, *Blk*, *Ccr2*, *Cd44*, *Cxcr6*, *Icos*, *Il1r1*, *Il23r*, *Klrbc1*, *Klrk1*, *Maf*, *Pdcd1*, *Rorc*, and *Tnfsf11*. This investigation showed that these genes are primarily localized to Clusters 1–6 (Fig. 5 B), reinforcing the notion that Clusters 1–6 represent CD27^−^ γδ T cells.

Having observed a dramatic increase in transcriptional diversity among lung γδ T cells from tumor-bearing KB1P mice, we investigated the gene expression differences between individual clusters. Of the 593 differentially expressed genes highlighted in the clustering analysis, 497 genes (84%) were shared between two or more clusters. We noticed that 315 of these shared genes were ribosomal-related genes (Table S2). After excluding these ribosome genes, we generated a heat map of the remaining 278 genes (Fig. 5 C) and we found that 184 genes (74%) were shared between two or more clusters. Clusters 3, 4, 5, and 7 were the most distinctive groups of cells, whereas Clusters 1, 2, and 6 were very similar to other clusters (Fig. 5 C). The genes unique to each individual cluster included 8 genes for Cluster 1, 0 genes for Cluster 2, 7 genes for Cluster 3, 22 genes for Cluster 4, 34 genes for Cluster 5, 2 genes for Cluster 6, and 21 genes for Cluster 7.

**Figure S3.**
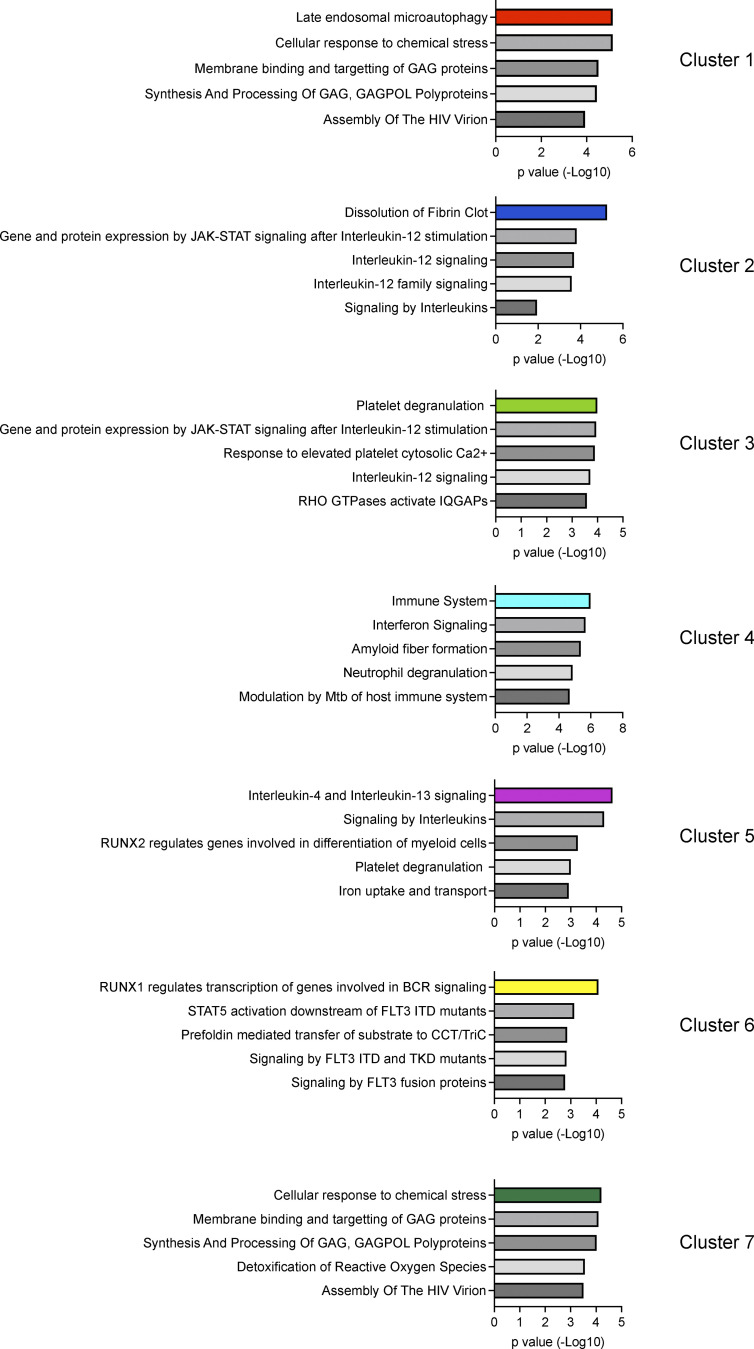
**Pathway analysis of scRNAseq clusters identified from γδ T cells in mammary tumor-bearing mice.** Differentially expressed genes for each individual cluster were analyzed by REACTOME pathway analysis. The top five pathways characterizing each cluster is shown. Group-specific antigen (GAG), DNA polymerase (POL), IQ motif-containing GTPase-activating proteins (IQGAPS), B cell receptor (BCR), internal tandem duplication (ITD), and tyrosine kinase domain (TKD).

Both shared and unique genes for each cluster were analyzed using REACTOME pathway analysis to gain a better understanding of how cluster-specific genes are related. Cluster 1 was defined by autophagy-related pathways. Clusters 2 and 3 were defined by IL-12 and JAK/STAT signaling pathways. Cluster 4 was defined by interferon signaling and neutrophil degranulation. Cluster 5 was defined by IL-4/IL-13 signaling, RUNX2 gene regulation, platelet degranulation as well as iron uptake and transport. Cluster 6 was defined by RUNX1 gene regulation and FLT3/STAT5 signaling. The CD27^+^ γδ T cell Cluster 7 was defined by cell responses to chemical stress, viruses, or reactive oxygen species (Fig. S3 and Table S3). Taken together, the data show that each cluster is transcriptionally distinct from the others, but overall, the clusters are very similar to each other, especially for Clusters 1–6.

Since Cluster 5 displayed the most unique set of genes, we determined what was driving these distinctive properties. Investigation of the Cluster 5 gene list revealed that *5830411N06Rik* (SCART2) and *Tcrg-C2*, two surrogate markers of Vγ4^+^ cells (Chen et al., 2019; Kisielow et al., 2008; Tan et al., 2019), are highly expressed (Fig. 5 D). We then used *Cd163l1* (SCART1) and *Tcrg-C1* to identify Vγ6^+^ cells, which showed that these cells are enriched in Clusters 1, 2, 3, 4, and 6, making up the majority of cells in the scRNAseq dataset. Vγ4^+^ and Vγ6^+^ cell markers were mostly absent from Cluster 7 (Fig. 5 D). To compare the transcriptional profile of Vγ4^+^ and Vγ6^+^ cells from lungs of KB1P tumor-bearing mice, *5830411N06Rik* and *Cd163l1* genes were used to distinguish the two subsets. Computational analysis revealed that there were 106 genes unique to *5830411N06Rik*-expressing and 46 genes unique to *Cd163l1*-expressing cells (Table S4). Among the differentially expressed genes, *5830411N06Rik*-expressing cells produced higher levels of *Cd48*, *Cd74*, *Fcer1g*, *Havcr2*, *Icos*, *Il17f*, *Irf4*, *Klf2*, *Lgals3*, *Ly6a*, and *Tcrg-C2*, while *Cd163l1*-expressing cells produced higher levels of *Bcl2a1a*, *Bcl2a1b*, *Cd3e*, *Cd69*, *Ifngr1*, *Ltb*, *Pdcd1*, *Tcrg-C1*, and *Tnf*. Moreover, *Il17a* was equally expressed between the two subsets (Fig. 5 E and Table S4). The gene signatures of the two γδ T cell subsets mostly corroborate observations made in skin Vγ4^+^ and Vγ6^+^ cells; although, there were some exceptions, such as the enrichment of *Ifngr1* in skin *5830411N06Rik*-expressing cells and lung *Cd163l1*-expressing cells (Tan et al., 2019). These dissimilarities between lung and skin cells may reflect the influence of tumor-derived factors on lung Vγ4^+^ and Vγ6^+^ cells. Indeed, lung Vγ4^+^ and Vγ6^+^ cells from tumor-bearing KB1P mice showed expression of *Havcr2*, *Icos*, *Ly6a*, and *Irf4*, for example (Fig. 5 E), which was not observed in cells from WT mice (Fig. 1).

### IL-1β and IL-23 drive the phenotypic diversity of lung γδ T cells

**Figure 6.**
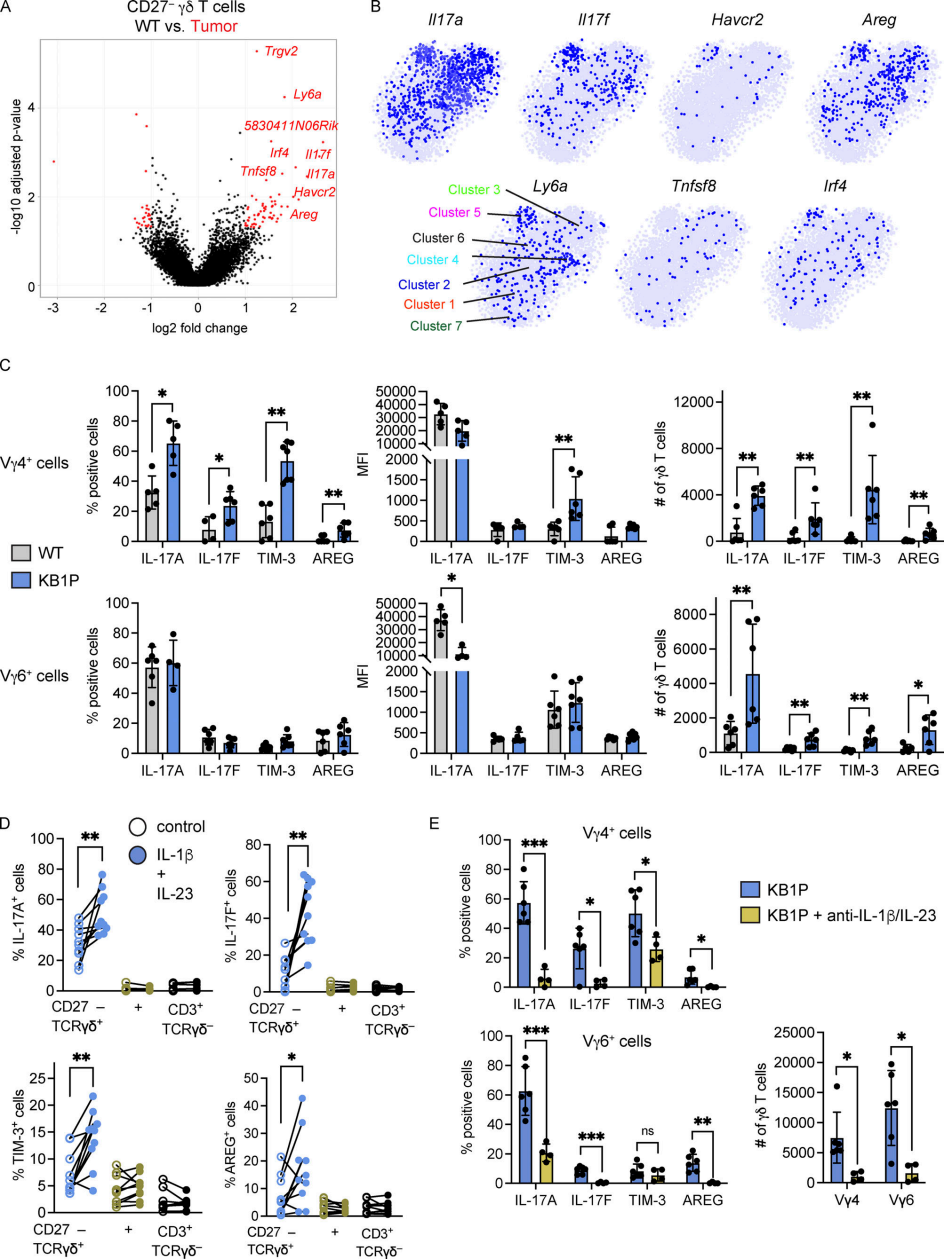
**Tumor-derived IL-1β and IL-23 drive gene expression and proliferation in lung γδ T cells. (A)** The transcriptome of CD27^—^ γδ T cells from KB1P Clusters 1–6 (Fig. 5 A) was compared to WT Cluster 1 (Fig. 1 A) and represented as a volcano plot. Genes up- or down-regulated in γδ T cells from KB1P Clusters 1–6 are denoted in red. **(B)** Feature plots in tSNE map of indicated genes from KB1P-derived γδ T cells. **(C)** Single-cell suspensions from lung of WT and tumor-bearing KB1P mice were stimulated for 3 h with PMA, ionomycin, and Brefeldin A. Cells were stained with antibodies against CD3, TCRδ, CD27, Vγ4, and Vγ6 to identify CD27^−^ Vγ4^+^ and Vγ6^+^ by flow cytometry. The proportion of cells expressing the indicated molecules, the MFI, and the absolute number of cells is represented graphically. Each dot represents one mouse. Data are presented as mean ± SD (*n* = 5–6 mice/group). Mann–Whitney U test; *P < 0.05, **P < 0.01. **(D)** CD3^+^ T cells were isolated from the lungs of FVB/n mice and stimulated with recombinant IL-1β and IL-23 for 15 h. Cells were stimulated for 3 h with PMA, ionomycin, and Brefeldin A and analyzed by flow cytometry. The proportion of cells expressing IL-17A, IL-17F, TIM-3, or AREG for each population indicated is shown. Each dot represents cell cultures from one mouse. Data are presented as mean ± SD (*n* = 10/group). Paired *t* test; *P < 0.05, **P < 0.01. **(E)** KB1P tumor-bearing mice were treated with blocking antibodies against IL-1β and IL-23 for three consecutive days. Control mice received non-specific antibodies. Lung CD27^−^ Vγ4^+^ and Vγ6^+^ cells were analyzed by flow cytometry 24 h after last injection. The proportion of cells expressing IL-17A, IL-17F, TIM-3, or AREG and the absolute numbers of each population is shown. Each dot represents one mouse. Data are presented as mean ± SD (*n* = 6 KB1P control, 4 KB1P + anti-IL-1β/IL-23). Mann-Whitney U test; *P < 0.05, **P < 0.01, ***P < 0.001.

How CD27^−^ γδ T cells react to tumor-derived factors beyond IL-17A is largely unknown. Therefore, we compared the transcriptome data of Cluster 1 from WT mice with Clusters 1–6 from tumor-bearing KB1P mice to determine which genes are differentially regulated in CD27^−^ γδ T cells by mammary tumors. A total of 96 genes, including *Il17a*, were upregulated in cells from tumor-bearing KB1P mice when compared with cells from WT mice, while 72 genes were downregulated (Fig. 6 A and Table S5). Among the upregulated genes, *5830411N06Rik* (SCART2), *Tcrg-C2*, and *Trgv2* were featured, indicating that Vγ4^+^ cells were more abundant in the lungs of tumor-bearing mice than tumor-free mice, which was also noted by flow cytometry (Fig. 4 B). Other upregulated genes included *Il17f*, *Havcr2* (TIM-3), *Areg* (amphiregulin), *Irf4*, *Ly6a* (SCA-1), and *Tnfsf8* (CD30L; Fig. 6, A and B). IL-17F, AREG, IRF4, and SCA-1 are frequently expressed by IL-17A–producing γδ T cells (Jin et al., 2019; Tan et al., 2019; Wang et al., 2021), and CD30L plays a role in maintenance of these cells (Sun et al., 2013). TIM-3 is a co-inhibitory molecule often expressed by CD8^+^ T cells or dendritic cells in the tumor microenvironment (de Mingo Pulido et al., 2021; Dixon et al., 2021; Wolf et al., 2020). We observed that *Il17f* and *Havcr2* expression were enriched in Cluster 5, where Vγ4^+^ cells were localized, while *Tnfsf8* was absent from Cluster 5 and the other genes were evenly distributed across Clusters 1–6 (Fig. 6 B).

**Figure S4.**
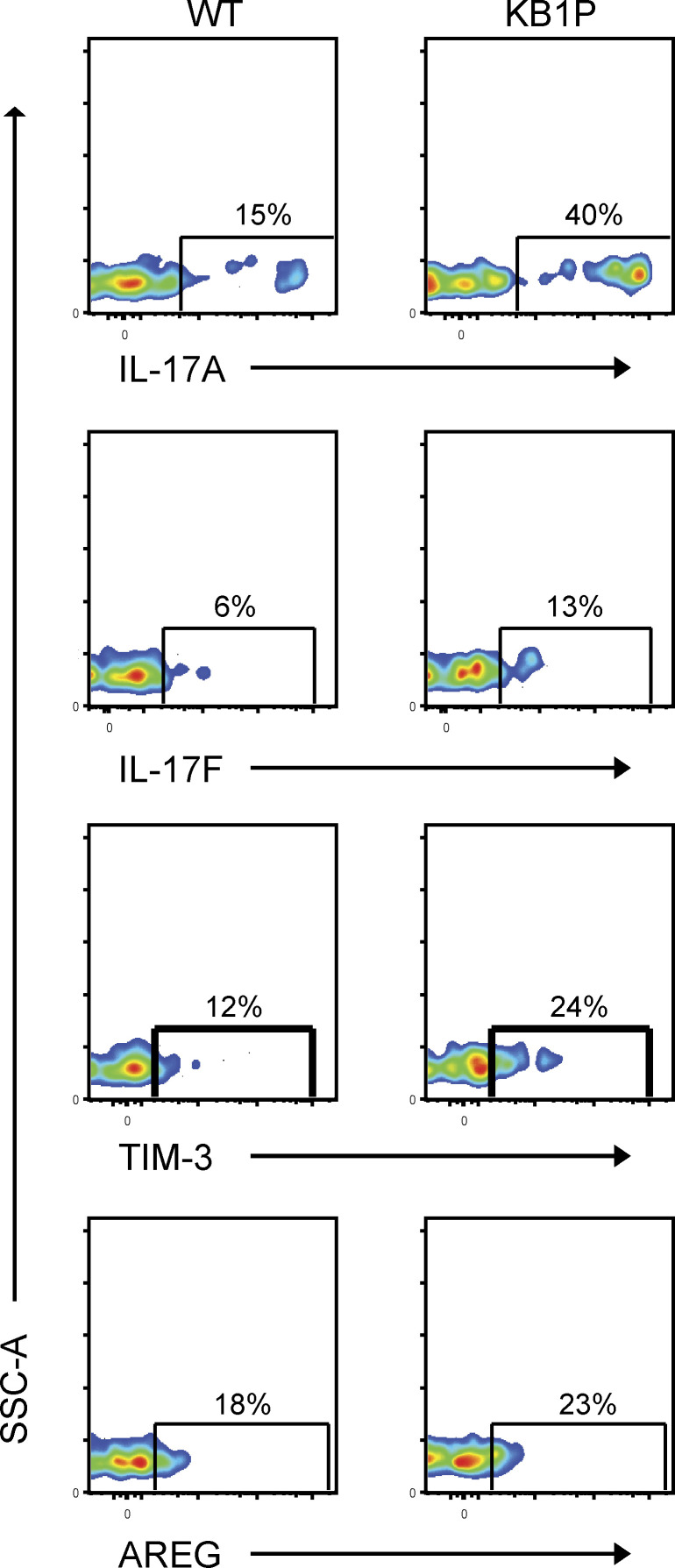
**Expression of IL-17A, IL-17F, TIM-3, and AREG by lung CD27**^**−**^ **γδ T cells.** Flow cytometry plots of staining for indicated molecules in lung CD27^−^ γδ T cells from WT and tumor-bearing KB1P mice. Single-cell suspensions of lung were stimulated for 3 h with PMA, ionomycin, and Brefeldin A, and stained for extracellular and intracellular markers and analyzed by flow cytometry.

We validated the upregulation of *Il17a*, *Il17f*, *Havcr2*, and *Areg* at protein level in Vγ4^+^ and Vγ6^+^ cells from the lungs of WT and tumor-bearing KB1P mice by flow cytometry. In Vγ4^+^ cells, the proportion of IL-17A, IL-17F, TIM-3, and AREG expression was increased in KB1P mice when compared to WT mice, whereas expression was similar for all proteins in Vγ6^+^ cells (Fig. 6 C and Fig. S4). The MFI of these proteins remained largely unchanged except for an increase in TIM-3 for Vγ4^+^ cells and decrease in IL-17A for Vγ6^+^ cells. The total number of all IL-17A–, IL-17F–, TIM-3–, and AREG-expressing Vγ4^+^ and Vγ6^+^ cells was increased in lungs of tumor-bearing mice when compared to tumor-free controls (Fig. 6 C), mirroring the increase of total cell numbers shown in Fig. 4 B. These data indicate that tumors drive expansion of Vγ4^+^ and Vγ6^+^ cells and that gene/protein expression in Vγ4^+^ cells is particularly responsive to tumor-derived factors, while the phenotype of Vγ6^+^ cells is mostly unaffected by tumor-derived factors.

Next, we determined how IL-17A, IL-17F, TIM-3, and AREG are regulated in CD27^−^ γδ T cells. In tumor-associated lungs, the systemic increase in IL-1β and IL-23 induces CD27^−^ γδ T cells to expand and rapidly produce cytokines (Coffelt et al., 2015; Jin et al., 2019). To recapitulate the systemic increase of IL-1β and IL-23 in mammary tumor-bearing mice, we stimulated CD3^+^ T cells isolated from lungs of WT mice in vitro with these cytokines and examined tumor-associated protein expression in CD27^−^ γδ T cells. This analysis revealed that IL-1β/IL-23 treatment increases expression of IL-17A, IL-17F, TIM-3, and AREG (Fig. 6 D). These increases were specific to the CD27^−^ γδ T cell population, as CD27^+^ γδ T cells and CD3^+^γδTCR^−^ T cells did not respond to IL-1β/IL-23 stimulation (Fig. 6 D). To further verify the influence of tumor-derived IL-1β and IL-23 on Vγ4^+^ and Vγ6^+^ cells in vivo, we treated KB1P mice bearing end-stage tumors with blocking antibodies against the cytokines. We observed that inhibition of IL-1β and IL-23 reduced the proportion of Vγ4^+^ cells expressing IL-17A, IL-17F, TIM-3, and AREG. The expression of IL-17A, IL-17F, and AREG, but not TIM-3, was also reduced in Vγ6^+^ cells by blocking IL-1β/IL-23 when compared to controls (Fig. 6 E). In addition, absolute numbers of Vγ4^+^ and Vγ6^+^ cells were reduced following IL-1β/IL-23 inhibition (Fig. 6 E). These findings indicate that the pro-inflammatory cytokines IL-1β and IL-23 drive the expansion and phenotype of pro-tumorigenic Vγ4^+^ and Vγ6^+^ cells in the lung of tumor-bearing mice.

### Tumor-associated γδ T cells expand with anti–PD-1 and anti–TIM-3 and confer resistance to checkpoint immunotherapy

**Figure 7.**
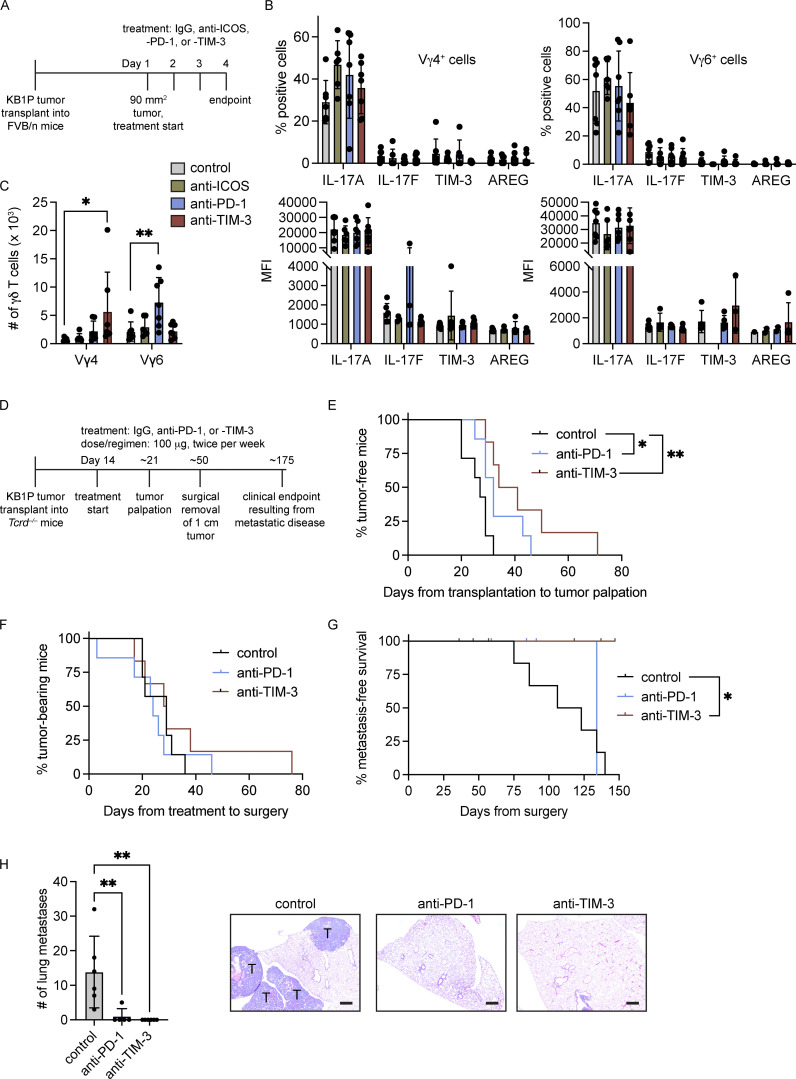
**Vγ4**^**+**^ **and Vγ6**^**+**^ **cells expand in response to anti–PD-1 and anti–TIM-3 and promote resistance to immunotherapy. (A)** Schematic of experimental procedure for orthotopic transplantation of KB1P tumor fragments into FVB/n mice and treatment with anti–PD-1, anti-ICOS, anti–TIM-3, or isotype control. **(B)** Single-cell suspensions from lung of tumor-bearing KB1P mice treated as indicated were stimulated for 3 h with PMA, ionomycin, and Brefeldin A. Cells were stained with antibodies to identify CD27^−^ Vγ4^+^ and Vγ6^+^ cells by flow cytometry. The proportion of cells expressing the indicated molecules and the MFI is represented graphically. Each dot represents one mouse. Data are presented as mean ± SD (*n* = 6–7 mice/group). One-way ANOVA followed by Dunnett’s posthoc test. **(C)** Absolute number of cells in lungs of KB1P tumor-bearing mice treated as indicated. Each dot represents one mouse. Data are presented as mean ± SD (*n* = 6–7 mice/group). One-way ANOVA followed by Dunnett’s posthoc test; *P < 0.05, **P < 0.01. **(D)** Schematic of experimental procedure for orthotopic transplantation of KB1P tumor fragments into *Tcrd*^*−/−*^ mice, treatment with anti–PD-1, anti–TIM-3, or isotype control, surgical removal, and clinical endpoint by metastatic disease (*n* = 6–7 mice/group). **(E)** Kaplan–Meier survival analysis of tumor formation in KB1P tumor-bearing mice treated as indicated (*n* = 7 control, 6 anti–PD-1, 6 anti–TIM-3). Log-rank test; *P < 0.05, **P < 0.01. **(F)** Kaplan-Meier survival analysis of tumor growth in KB1P tumor-bearing *Tcrd*^*−/−*^ mice treated as indicated (*n* = 7 control, 6 anti–PD-1, 6 anti–TIM-3). Log-rank test. **(G)** Kaplan-Meier survival analysis of metastasis formation in KB1P tumor-bearing *Tcrd*^*−/−*^ mice treated as indicated. Mice that developed local recurrence at the primary tumor site after surgery are censored (*n* = 6 control, 5 anti–PD-1, 6 anti–TIM-3). Log-rank test; *P < 0.05. **(H)** Quantification of lung tumors in KB1P tumor-bearing *Tcrd*^*−/−*^ mice treated as indicated with representative images of lung sections. T = tumor nodules. Each dot represents one mouse. Data are presented as mean ± SD (*n* = 6 control, 5 anti–PD-1, 6 anti–TIM-3). One-way ANOVA followed by Dunnett’s posthoc test; **P < 0.01. Scale bar = 60 μm.

**Figure S5.**
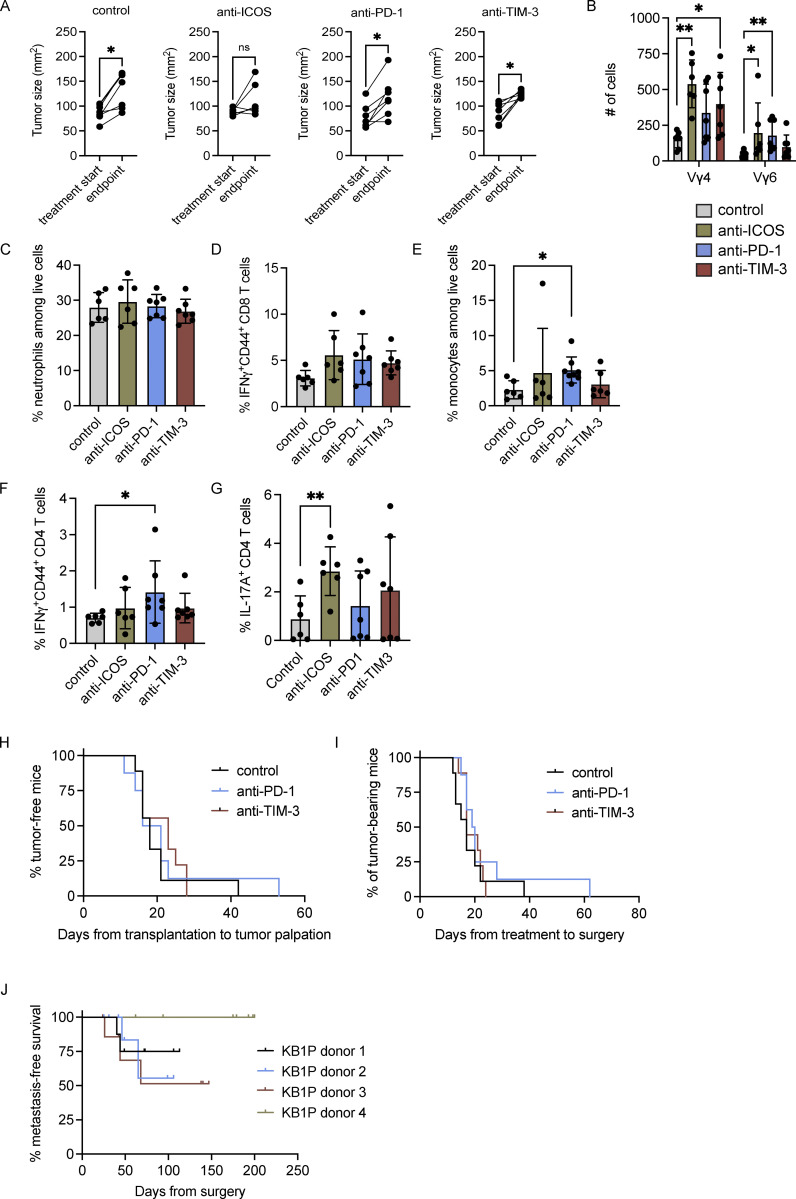
**Effects of anti-ICOS, –PD-1, and –TIM-3 on tumor growth and immune cell phenotype. (A)** Graphic representation of transplanted KB1P tumor growth at start of treatment and experimental endpoint in mice treated as indicated. Each dot represents one mouse. Data are presented as mean ± SD (*n* = 6 mice per group). *P < 0.05 as determined by paired t test. **(B)** Absolute number of cells in lungs of KB1P tumor-bearing mice treated as indicated. Each dot represents one mouse. Data are presented as mean ± SD (*n* = 6–7 mice/group). One-way ANOVA followed by Dunnett’s posthoc test; *P < 0.05, **P < 0.01. **(C)** Percentage of circulating neutrophils in isotype control, anti–PD-1-, anti-ICOS–, and anti–TIM-3-treated tumor-bearing mice as measured by IDEXX ProCyte hematology analyzer. Each dot represents one mouse. Data are presented as mean ± SD (*n* = 6–7 mice/group). One-way ANOVA followed by Dunnett’s posthoc test. **(D–G)** Percentage of indicated populations from lungs of isotype control, anti–PD-1-, anti-ICOS–, and anti–TIM-3-treated KB1P tumor-bearing mice as measured by flow cytometry. Each dot represents one mouse. Data are presented as mean ± SD (*n* = 6–7 mice/group). One-way ANOVA followed by Dunnett’s posthoc test; *P < 0.05, **P < 0.01. **(H)** Kaplan-Meier survival analysis of tumor formation in KB1P tumor-bearing WT mice treated as indicated (*n* = 9 control, 8 anti–PD-1, 9 anti–TIM-3). Log-rank test. **(I)** Kaplan–Meier survival analysis of tumor growth in KB1P tumor-bearing WT mice treated as indicated (*n* = 9 control, 8 anti–PD-1, 9 anti–TIM-3). Log-rank test. **(J)** Kaplan–Meier survival analysis of metastasis formation in four different KB1P donor tumors transplanted into WT mice (*n* = 8 donor 1, 9 donor 2, 8 donor 3, 9 donor 4). Mice that developed local recurrence at the primary tumor site after surgery are censored.

Having established the effects of tumor-derived factors on Vγ4^+^ and Vγ6^+^ cells as well as the differential expression of the T cell checkpoint molecules, PD-1 and TIM-3, on these cells, we set out to determine how inhibition of these checkpoint molecules may influence Vγ4^+^ and Vγ6^+^ cells in the pre-metastatic niche. We used the KB1P tumor model to condition lung Vγ4^+^ and Vγ6^+^ cells. KB1P tumor fragments were transplanted into the mammary glands of syngeneic mice and allowed to grow to ∼1 cm. After this, mammary tumor–bearing mice received daily doses of anti-ICOS, anti–PD-1, or anti–TIM-3 over 3 d to mitigate the confounding effects of these antibodies on other T cell subsets (Fig. 7 A). Tumor growth increased in control, anti–PD-1-, and anti–TIM-3-treated mice, whereas tumor growth was prevented in anti-ICOS–treated mice (Fig. S5 A). Analysis of lung Vγ4^+^ and Vγ6^+^ cells in mammary tumor-conditioned lungs revealed that inhibition of ICOS, PD-1, or TIM-3 failed to alter the proportion of cells expressing IL-17A, IL-17F, TIM-3, or AREG. MFI of these molecules was also unaffected (Fig. 7 B). We then calculated the absolute numbers of Vγ4^+^ and Vγ6^+^ cells, since anti–PD-1 increased Vγ6^+^ cells in tumor-free mice (Fig. 3 B). Here, we observed subset-specific responses to the checkpoint inhibitors. Anti–TIM-3 treatment of KB1P tumor-bearing mice (but not other checkpoint inhibitors) increased Vγ4^+^ cell numbers when compared with control-treated mice, while anti–PD-1 treatment (but not other checkpoint inhibitors) increased Vγ6^+^ cell numbers when compared with control (Fig. 7 C). This contrasting response by Vγ4^+^ and Vγ6^+^ cells to distinctive checkpoint inhibitors accords well with their opposing expression of PD-1 and TIM-3. In the lymph node of these KB1P tumor-bearing mice, we found a similar response. Anti–TIM-3 increased Vγ4^+^ cell numbers, while anti–PD-1 treatment increased Vγ6^+^ cell numbers when compared with control. Interestingly, anti-ICOS treatment increased both Vγ4^+^ and Vγ6^+^ cells (Fig. S5 B), although it is not clear why lymph node cells respond in this way when lung cells do not. These data indicate that the proliferative effects of anti–PD-1 and anti–TIM-3 on Vγ4^+^ and Vγ6^+^ cells extend beyond the lung.

IL-17A–producing γδ T cells promote metastasis through expansion of neutrophils which in turn inhibit CD8^+^ T cells so that disseminated cancer cells subvert anti-tumor immunity (Coffelt et al., 2015; Wellenstein et al., 2019). Therefore, neutrophil frequency and the phenotype of conventional T cells was investigated in tumor-bearing KB1P mice treated with anti–PD-1, anti–TIM-3, or anti-ICOS, as a systemic readout of increased IL-17A by γδ T cells. However, neutrophil frequency in blood as well as expression of CD44 and IFNγ by lung CD8^+^ T cells remained the same between treatment groups (Fig. S5, C and D), suggesting that the increased IL-17A in this short-term experiment fails to reach a threshold large enough to stimulate granulopoiesis and elevate circulating neutrophils or suppress CD8^+^ T cells. Unexpectedly, we observed increased proportions of monocytes and IFNγ^+^CD44^+^ CD4^+^ T cells in anti–PD-1-treated KB1P tumor-bearing mice (Fig. S5, E and F). In addition, anti-ICOS treatment of tumor-bearing mice increased IL-17A expression by CD4^+^ T cells in the lung (Fig. S5 G). Overall, we found that acute inhibition of ICOS, PD-1, and TIM-3 modulates γδ T cells and various other immune cells in different ways.

Given the proliferative effect of anti–PD-1 and anti–TIM-3 on pro-metastatic Vγ6^+^ and Vγ4^+^ cells, respectively, we hypothesized that increased numbers of IL-17A–producing γδ T cells may counteract checkpoint inhibitor activation of CD8^+^ T cells in immunotherapy-treated mice. The KB1P model is inherently resistant to anti–PD-1 therapy (Blatter et al., 2018; Hollern et al., 2019), making it an appropriate model to test this hypothesis. We confirmed that KB1P transplanted tumors are refractory to checkpoint inhibitors, as neither anti–PD-1 nor anti–TIM-3 changed the rate of tumor initiation or tumor growth (Fig. S5, H and I). We then transplanted KB1P fragments into γδ T cell–deficient (*Tcrd*^*−/−*^) mice and treated these mice as shown in Fig. 7 D. We measured tumor initiation, tumor growth, and metastasis formation in these *Tcrd*^*−/−*^ mice. We found that the time from transplantation to tumor initiation was delayed in tumor-bearing mice treated with either anti–PD-1 or anti–TIM-3 immunotherapy (Fig. 7 E), suggesting that the absence of γδ T cells unleashes checkpoint inhibitor-activated anti-tumor immunity in a model that is normally refractory to immunotherapy. By contrast, the checkpoint inhibitors had no impact on tumor growth in *Tcrd*^*−/−*^ mice (Fig. 7 F). After surgical removal of tumors, mice were monitored for metastatic disease, which presents in the form of respiratory distress caused by lung tumor burden. Mice that died from causes other than metastasis (usually recurrence at the surgical site) were censored. KB1P mammary tumors do not readily spread to lungs in WT mice (Fig. S5 J). However, all *Tcrd*^*−/−*^ mice treated with control isotype antibody (6 out of 6) succumbed to metastatic disease, whereas only 1 out of 5 *Tcrd*^*−/−*^ mice treated with anti–PD-1 and 0 out of 6 *Tcrd*^*−/−*^ mice treated with anti–TIM-3 developed lung metastases (Fig. 7 G). Histological analysis of lungs from *Tcrd*^*−/−*^ mice confirmed that 100% of the control group contained secondary tumors. By contrast, only one lung from the anti–PD-1 group and zero from the anti–TIM-3 group contained secondary tumors (Fig. 7 H). Taken together, these data show that genetic deletion of IL-17A–producing Vγ6^+^ and Vγ4^+^ cells sensitizes metastatic mammary cancer cells to checkpoint inhibitor immunotherapy.

## Discussion

IL-17A–producing γδ T cells within the lung consist mostly of Vγ4^+^ and Vγ6^+^ cells that can function as either protective or pathogenic cells during infection, inflammation, and allergy (Faustino et al., 2020; Guo et al., 2018; Misiak et al., 2017; Wang et al., 2021). These cells are also key drivers of lung cancer and pulmonary metastasis (Coffelt et al., 2015; Jin et al., 2019; Kulig et al., 2016). Here, we used scRNAseq with protein validation to provide novel insight into IL-17A–producing γδ T cell subsets in normal and tumor-conditioned lung. Our data show that Vγ6^+^ cells express a phenotype with high degree of similarity to T_rm_ CD8^+^ T cells through production of CXCR6, ICOS, JAML, and PD-1, whose expression is stable between tumor-free and tumor-bearing mice. Both Vγ6^+^ and Vγ4^+^ cells rapidly expand in response to tumor-derived factors, such as IL-1β and IL-23. These cytokines induce TIM-3 specifically on Vγ4^+^ cells in tumor-bearing mice. The constitutive expression of PD-1 on Vγ6^+^ cells and inducible expression of TIM-3 on Vγ4^+^ cells has revealed the differential control of these discrete subsets by distinct co-inhibitory molecules.

Our data add to a growing body of literature centered around the expression and function of co-stimulatory and co-inhibitory molecules on IL-17A–producing γδ T cells. In agreement with our data, other studies have reported high levels of ICOS and PD-1 expression on Vγ6^+^ cells from the uterus and skin (Monin et al., 2020; Tan et al., 2019), suggesting that these two molecules are common features of Vγ6^+^ cells in any tissue. PD-1–deficient mice exhibit elevated levels of IL-17A from γδ T cells and greater disease severity of imiquimod-driven psoriasis (Imai et al., 2015; Kim et al., 2016), which accords well with our observations. We show that PD-1 signaling can suppress Vγ6^+^ cell expansion; however, the role of ICOS on Vγ4^+^ and Vγ6^+^ cells is unclear. The ICOS/ICOS-ligand (ICOSL) axis is critical for the development of IL-17A–producing γδ T cells. ICOS-deficient mice develop increased numbers of Vγ4^+^ cells than WT controls, and experimental autoimmune encephalomyelitis is more severe in these mice as a result of increased IL-17A levels (Buus et al., 2016; Galicia et al., 2009). Similar observations were made in lymph nodes of B and T lymphocyte attenuator (BTLA)–deficient mice where Vγ4^+^ cells expand and produce more IL-17A (Bekiaris et al., 2013). Whether the development of Vγ6^+^ cells is affected by loss of ICOS or BTLA remains unknown. For Vγ4^+^ cells, our data show that TIM-3 expression is positively regulated by the cytokines IL-1β and IL-23. It seems that TIM-3 is not the only co-inhibitory receptor that is inducible on Vγ4^+^ cells, as BTLA, CTLA-4, and PD-1 are also increased by IL-1β, IL-23, or IL-7 stimulation (Bekiaris et al., 2013; Kadekar et al., 2020). Taken together, these observations have important implications for the regulation of discrete γδ T cell subsets during homeostasis and disease.

One interesting question to arise from these data is how PD-1 and TIM-3 signaling inhibits proliferation of Vγ6^+^ and Vγ4^+^ cells. In conventional T cells, PD-1 signaling through SHP-1 and SHP-2 phosphatases disrupt TCR signaling and CD28 co-stimulation through the inhibition of LCK and ZAP70 activation of PI3K/AKT and MAPK pathways (Sharpe and Pauken, 2018). TIM-3 binds an adaptor protein called BAT3 (or BAG6) that negatively regulates its function (Rangachari et al., 2012; Wolf et al., 2020). Activation of TIM-3 by Galectin-9 or another ligand releases its interaction with BAT3, which leads to suppression of mTORC2 and AKT phosphorylation downstream of TCR signaling, as well as nuclear localization of the transcription factor FOXO1 (Zhu et al., 2021). However, activation of IL-17A–producing γδ T cells is generally considered independent of TCR signaling—these cells are stimulated by cytokines rather than antigen. Moreover, our data show that PD-1 inhibition of Vγ6^+^ cells is independent of STAT3, NF-κB, AKT, and MAPK signaling pathways. Therefore, inhibitory pathways induced by PD-1 and TIM-3 are different between αβ and γδ T cells. We found that FOXO1 phosphorylation is modulated by PD-1 signaling in Vγ6^+^ cells, but the mechanism of FOXO1 phosphorylation remains a mystery since AKT activity was unaltered by PD-1–PD-L1 engagement. How FOXO1 regulates Vγ6^+^ cell behavior is also unknown. Three possibilities stand out from the literature. First, FOXO1 is an inhibitor of the IL-17A master regulator, RORγt (Lainé et al., 2015), so interaction between these two transcription factors may alter cell cycle. Second, FOXO1 controls expression of p27 to repress cell proliferation (Medema et al., 2000). Third, FOXO1 regulates glucose and lipid metabolism (Ma et al., 2018). Given that IL-17A–producing γδ T cells are dependent on lipid metabolism (Lopes et al., 2021), FOXO1 downstream of PD-1 may affect the fitness of Vγ6^+^ cells. FOXO1 phosphorylation was not altered in Vγ4^+^ cells, so how TIM-3 signaling inhibits these cells remains unknown. Further experimentation is required to fully elucidate the mechanism of PD-1 and TIM-3 suppression of Vγ6^+^ and Vγ4^+^ cells.

Another consideration to the regulation of Vγ4^+^ and Vγ6^+^ cells in the lung (or other tissues) is their spatial location, tissue compartmentalization, and interaction with other cells at these sites. A previous study has shown that Vγ4^+^ cells are mainly found in lung parenchyma, whereas putative Vγ6^+^ cells are mostly found in non-parenchymal locations (Wands et al., 2005). In addition, a large proportion of lung γδ T cells interact with myeloid cells, which coincidentally are among the largest producers of PD-L1 and TIM-3 ligands, such as Galectin-3. Identifying which cells meet Vγ4^+^ and Vγ6^+^ cells during infection, inflammation, or cancer progression will provide important insight into their behavior.

There is evidence from clinical studies to suggest that IL-17A signaling undermines immune checkpoint inhibitors, contributes to immunotherapy resistance, and promotes the development of adverse autoimmune events in cancer patients (Kang et al., 2021). As such, modulation of PD-1 and TIM-3 in cancer patients and the impact of inhibiting these molecules on γδ T cells is a point for consideration, as these immunotherapy drugs may deleteriously increase IL-17A expression and immunosuppressive neutrophil activation. Indeed, immune checkpoint inhibitor non-responding melanomas contain a greater proportion of a γδ T cell subset than responding melanomas, and a gene signature derived from these γδ T cells can predict response to immune checkpoint inhibitors (Xiong et al., 2020). The effect of immune checkpoint inhibitors on IL-17A is not limited to γδ T cells, as human CD4^+^ T cells increase IL-17A after anti–PD-1 exposure (Bandaru et al., 2014) and IL-17A–producing cells in general are associated with anti–PD-1 resistance in melanoma, lung, and colorectal cancer (Gopalakrishnan et al., 2018; Li et al., 2021; Llosa et al., 2019; Peng et al., 2021). In agreement with our data, anti–PD-1 treatment of an autochthonous lung cancer mouse model increases IL-17A–producing γδ T cells and CD4^+^ T cells, which correlates with suppression of cytotoxic CD8^+^ T cells (Li et al., 2021). Moreover, IL-17A–overexpressing lung tumors in mice are resistant to anti–PD-1 therapy (Akbay et al., 2017). However, inhibition of IL-17A in mice bearing KRAS-mutant lung tumors or transplantable colon cancer cell lines has provided proof-of-principle that targeting IL-17A in combination with anti–PD-1 is a viable strategy for controlling tumor growth (Li et al., 2021; Liu et al., 2021; Peng et al., 2021). Anti–PD-1 immunotherapy, although effective against some melanomas, can also exacerbate psoriasis and colitis (Tanaka et al., 2017), but IL-17A blockade may reverse these toxicities (Esfahani and Miller, 2017; Johnson et al., 2019). Taken together, our observations reported herein and studies in the literature suggest that targeting IL-17A in combination with immune checkpoint inhibitors may thwart resistance mechanisms and lessen adverse autoimmune events in cancer patients.

## Materials and methods

### Mice

Female FVB/n mice (10–12 wk old) were used throughout the study. These mice were bred at the Cancer Research UK Beatson Institute from the *K14-Cre*;*Brca1*^*F/F*^;*Trp53*^*F/F*^ (KB1P) colony (Liu et al., 2007), which was gifted from Jos Jonkers (Netherlands Cancer Institute, Amsterdam, Netherlands). After arrival from the Jonkers lab, KB1P mice were backcrossed onto the FVB/N background for six generations. *Cre* recombinase negative mice were used as WT sources for γδ T cell phenotyping and scRNA analysis. *Cre* recombinase positive mice were monitored twice weekly for tumor formation by palpation and caliper measurements starting at 4 mo of age. γδ T cells in tumor-bearing KB1P mice were analyzed when tumors reached >1.2 cm; these mice were typically 7–8 mo old. Female C57BL/6J mice (10–12 wk old) were also bred in-house, and these mice were WT for every allele. Female *Tcrb*^*−/−*^ mice on C57BL/6J background (purchased from Jackson Labs) were bred in-house. For KB1P tumor transplantation studies, female FVB/n mice (6–8 wk old) were purchased from Charles River. Purchased mice were allowed to acclimatize before procedure until 10 wk old. Transplantation of tumor fragments was performed as previously described (Millar et al., 2020). Once tumors reached 1 cm, mice were randomized into control or experimental groups. All animals were bred under specific pathogen–free conditions in individually ventilated cages with unrestricted access to food and water. Procedures were performed in accordance with UK Home Office license numbers 70/8645 and PP6345023 (to Karen Blyth), and they were carried out in line with the Animals (Scientific Procedures) Act 1986 and the EU Directive 2010 and sanctioned by Local Ethical Review Process (University of Glasgow).

For short-term antibody inhibition studies, WT female FVB/n mice (Charles River) aged 10–12 wk, with or without transplanted KB1P tumors, were injected intraperitoneally with a single dose of 200 μg anti–PD-1 (clone RMP1-14; BioXcell), anti-ICOS (clone 7E.17G9; BioXcell), or anti–TIM-3 (clone RMT3-23; BioXcell), followed by single injections of 100 μg antibody on two consecutive days. Control mice followed the same dosage regime with isotype control (Rat IgG2a or Armenian Hamster IgG, BioXcell). We used the same treatment regimen and dosage for spontaneous KB1P tumor-bearing mice given anti–IL-1β (clone B122; BioXcell) and anti–IL-23 (clone G23-8; BioXcell). Mice were sacrificed 24 h after the third antibody injection.

For long-term antibody inhibition studies in our spontaneous metastasis model, WT female FVB/n mice (Charles River) or *Tcrd*^*−/−*^ mice on FVB/n background (gifted to us from Adrian Hayday, Francis Crick Institute, London, UK) were transplanted with KB1P tumor fragments as described (Millar et al., 2020). Mice were injected intraperitoneally with 100 μg anti–PD-1 (clone RMP1-14; BioXcell) or anti–TIM-3 (clone RMT3-23; BioXcell) twice per week, beginning 2 wk after tumor fragment transplantation until humane endpoint. Control mice followed the same dosage regime with isotype control. Tumor growth was monitored three times per week by calipers. Once tumors reached 1 cm, surgery was performed to excise the tumor. Following tumorectomy, mice were monitored daily for signs of tumor recurrence at the surgical site and respiratory distress due to lung tumor burden. Mice that developed recurrences before signs of respiratory distress were censored from survival curves.

### Tissue processing

Lungs were mechanically dissociated using a scalpel and transferred to collagenase solution consisting of DMEM medium (Thermo Fisher Scientific) supplemented with 1 mg/ml collagenase D (Roche) and 25 µg/ml DNase 1 (Thermo Fisher Scientific). Enzymatic dissociation was carried out using the gentleMACS Octo Dissociator, run: 37C_m_LDK_01 (Miltenyi Biotec), as described (Curio et al., 2022). Lung suspensions were filtered through a 70-µm cell strainer, using a syringe plunger, and enzyme activity was stopped by addition of 2 ml FCS followed by 5 ml DMEM medium supplemented with 10% FCS, 2 mM L-glutamine (Thermo Fisher Scientific), and 10,000 U/ml penicillin/streptomycin (Thermo Fisher Scientific). Lymph nodes were forced through a 70-µm cell strainer, using a syringe plunger, and the tissue was flushed through with PBS containing 0.5% BSA. Blood was drawn after cardiac puncture and collected in EDTA tubes and kept at room temperature for hematology analysis by Idexx (ProCyte Dx Haematology Analyzer) for monocyte and neutrophil quantification. For red blood cell lysis, the cell pellets were resuspended in 5 ml of commercially available 1× Red Blood Cell Lysis buffer (Thermo Fisher Scientific) for 3 min. Cells were resuspended in PBS containing 0.5% BSA and cell number was acquired, using a hemocytometer.

### scRNAseq

Total γδ T cells were sorted from lungs of eight littermate mice (four *Cre* negative and four tumor-bearing KB1P mice) from the KB1P colony by gating on DAPI^−^CD3^+^TCRδ^+^ cells (Fig. S1). Typically, 5,000 γδ T cells were captured from one lung, using a BD FACSAria II Cell Sorter. The sorted cells were loaded onto the Chromium Single Cell 30 Chip Kit v2 (10×Genomics) to generate libraries for scRNAseq, following the manufacturer’s instructions. The sequencing-ready library was cleaned up with SPRIselect beads (Beckman Coulter). Quality control of the library was performed prior to sequencing (Qubit, Bioanalyzer, quantitative PCR). Illumina sequencing was performed using NovaSeq S1 by Edinburgh Genomics (University of Edinburgh). The output .bcl2 file was converted to FASTQ format using cellranger-mkfastq algorithm (10×Genomics), and cellranger-count was used to align to the mm10 reference murine genome and build the final (cell,  unique molecular identifier) expression matrix for each sample. After quality control for removal of cells with >3,000 or <200 genes, and cells with more that 10% of reads from mitochondrial genes, followed by cell cycle correction, we obtained single-cell transcriptomes from 3,796 γδ T cells from lungs of WT mice and 5091 γδ T cells from lungs of mammary tumor–bearing KB1P mice. The data were normalized with the Seurat V3 “LogNormalize” method and batch effects were corrected with the Batchelor package. Then, principal component analysis and unsupervised clustering were performed on the data using Seurat. The first 20 principal components were retained for dimensional reduction and tSNE was utilized for visualization of the data. Marker genes and differentially expressed genes of cell clusters were determined using Seurat and DESeq2, respectively. The signature scores were visualized as heatmap projected on the dataset tSNE, with contours around those cells scoring >superior quartile. Raw data are deposited at ArrayExpress (#E-MTAB-10677). Gene signatures defining clusters were analyzed by Reactome pathway analysis (http://reactome.org).

### Flow cytometry

Single-cell suspensions were added to 96-well V bottom plates at a maximum density of 4 × 10^6^ cells/well. Cells were stimulated for 3 h at 37°C with complete IMDM medium with 1× Cell Activation Cocktail (with Brefeldin A, Biolegend). After stimulation, cells were centrifuged at 800 *g* for 2 min. Cells were incubated in blocking buffer (50 µl PBS/0.5% BSA, 1 µl FcR block [Biolegend]) for 20 min at 4°C. Antibodies for surface antigens were prepared in Brilliant stain buffer (BD Biosciences) and cells were stained for 30 min at 4°C in the dark. Cells were washed with PBS/0.5% BSA, centrifuged at 800 *g* for 2 min, followed by ice-cold PBS, and incubated with Zombie NIR Fixable Viability dye (Biolegend) to exclude dead cells for 20 min at 4°C. After washing the cells with PBS/0.5% BSA, cells were fixed and permeabilized in FOXP3 Transcription Factor Fixation/Permeabilization solution (Thermo Fisher Scientific) for 20 min at 4°C, following the manufacturer’s instructions. Intracellular antibodies were prepared in permeabilization buffer and cells were incubated for 30 min at 4°C. Cells were washed with permeabilization buffer, followed by PBS/0.5% BSA, and resuspended in PBS/0.5% BSA. Fluorescence minus one control samples (where only one antibody is left out of the panel) were prepared from spare cells to discriminate positive and negative staining. All experiments were performed using a five-laser BD LSRFortessa flow cytometer with DIVA software (BD Biosciences). Compensation was determined automatically using Ultracomp eBeads (#01-2222-42; Thermo Fisher Scientific). Data were analyzed using FlowJo Software version 9.9.6 or 10.8.1. Antibodies utilized for analysis are listed in Table 1.

**Table 1.** Antibodies utilized for flow cytometry

Antigen	Conjugate	Clone	Source	Catalogue	Dilution
AREG	Biotin—strepBV711	BAF989	Roche	BAF989	1:50
BATF	PE	MBM7C7	Thermo Fisher Scientific	12-9860-42	1:50
CD11b	APC-eFluor780	M1/70	eBioscience	47-0112-82	1:800
CD19	APC-eFluor780	1D3	eBioscience	47-0193-82	1:400
CD27	PE/Dazzle594	LG.3A10	BioLegend	124228	1:400
CD27	BV510	LG.3A10	BD	563605	1:200
CD3	BV650	17A2	BioLegend	100229	1:200
CD30L	PE	RM153	BioLegend	106405	1:200
CD4	APC-eFluor780	GK1.5	Invitrogen	47-0041-82	1:200
CD44	PerCP-Cy5.5	IM7	BioLegend	103032	1:100
CD8A	APC-eFluor780	53-6.7	Invitrogen	47-0081-82	1:100
CD8A	BUV805	53-6.7	BD	564920	1:200
CXCR6	BV711	SA051D1	BioLegend	151111	1:200
DAPI	N/A	N/A	Sigma-Aldrich	D9542	1 mg/ml
EpCAM	APC-eFluor780	G8.8	eBioscience	47-5791-82	1:50
Phospho-FOXO1	N/A		Cell Signaling	9464	1:50
ICOS	PE-Cy7	7E.17G9	Thermo Fisher Scientific	313519	1:50
IFNγ	PE-Cy7	XMG1.2	eBioscience	25-7311-82	1:200
IL-17A	PE	eBio17B7	eBioscience	12-7177-81	1:200
IL-17A	APC	eBio17B7	eBioscience	17-7177-81	1:200
IL-17F	PerCP-Cy5.5	079-289	BioLegend	562194	1:100
IRF4	PE-Cy7	IRF4.3E4	BioLegend	646413	1:100
JAML	A647	4E10	BioLegend	128506	1:50
NK1.1	BV421	PK136	BioLegend	108731	1:100
NKG2D	PE/Dazzle594	CX5	BioLegend	130214	1:100
NKG2D	APC	CX5	eBioscience	17-5882-82	1:200
PD-1	BV510	29F.1A12	BioLegend	135241	1:100
PD-1	PE-Cy7	29F.1A12	BioLegend	135215	1:100
RANKL	PE	IK22/5	BioLegend	510005	1:25
RORγt	PE-Cy7	B2D	Thermo Fisher Scientific	25-6981-82	1:100
SCA-1	BV605	D7	BioLegend	108133	1:100
Streptavidin	BV711	N/A	BioLegend	405241	1:100
TCR Vγ1	PE	2.11	Biolegend	141106	1:200
TCR Vγ4	APC	UC3-10A6	BioLegend	137707	1:100
TCR Vγ6	Pacific Blue	1C10-1F7	Hatano et al., 2019	N/A	1:50
TCRδ	FITC	GL3	eBioscience	11-5711-85	1:200
TIM-3	PE/Dazzle594	B8.2C12	BioLegend	134013	1:200
Zombie NIR	N/A	N/A	BioLegend	423106	1:400
Anti-rabbit IgG-biotin	N/A		Sigma-Aldrich	SAB4600006	1:1,000

### In vitro T cell stimulation

CD3^+^ T cells from lung single-cell suspensions (1 × 10^7^ cells) harvested from *Cre* negative KB1P FVB/n or *Tcrb*^*−/−*^ mice on C57BL/6J background were isolated by negative selection using the MojoSort mouse CD3^+^ T-cell Isolation kit (#480031; Biolegend) according to the manufacturer’s instructions. γδ T cell isolation was performed by TCRγδ isolation kit (#130-092-125; Miltenyi) according to the manufacturer’s instructions. Cells after enrichment were counted with a hemocytometer and plated at a density of 1 × 10^6^ CD3^+^ T cells/ml or 2–3.5 × 10^5^ γδ T cells/ml in round-bottomed 96-well plates in IMDM medium, 10% FCS, 2 mM L-glutamine, 10,000 U/ml penicillin/streptomycin, and 50 μM β-mercaptoethanol. Where indicated, wells were coated with 6 μg PD-L1-Fc chimera protein (R&D Systems) or anti-ICOS (clone C398.4A; Biolegend) in PBS overnight at 4°C. T cells were cultured in the presence of these antibodies for 3, 15, or 24 h as indicated. Cells were stimulated for last 30 min of incubation with 2.5 ng/ml recombinant IL-1β (ImmunoTools) and IL-23 (R&D Systems).

### ELISA and Western blot

IL-17A levels in conditioned medium from lung, CD3^+^ T cell cultures were determined by ELISA after 24 h stimulation (R&D Systems) according to the manufacturer’s instructions. Individual groups were tested in triplicate for technical replicates. Each experiment was repeated at least five times.

For Western blot, cell suspensions were harvested directly into pre-cooled Eppendorf tubes rinsed twice with ice-cold PBS. The collected cell suspensions were spun at 6,000 rpm for 2 min and supernatants were completely removed. Protein was extracted from each tube and entire contents resolved on 4–12% NuPage polyacrylamide gels essentially as described (Millar et al., 2020). Primary antibodies were incubated overnight at 4°C on blocked membranes. Antibodies not previously described include anti-phospho-STAT3 (1:2,000; #9145; Cell Signaling Technology), anti-STAT3 (1:1,000; #9139; Cell Signaling Technology), anti-phospho-p65 (1:1,000; #3033; Cell Signaling Technology), anti-p65 (1:1,000; #8242; Cell Signaling Technology), anti-phospho-FOXO1/FOXO3A (1:1,000; #9464; Cell Signaling Technology), and anti-FOXO1 (1:1,000; #18592-1-AP; Proteintech). HRP-linked secondary antibodies (Cell Signaling Technology) were used at 1:2,000 as described (Millar et al., 2020). Proteins were visualized by chemiluminescence using the ChemiDoc system (Bio-Rad).

### Quantification of lung metastases

Lung sections from FVB/n mice transplanted with KB1P tumor fragments and treated with anti–PD-1 or anti–TIM-3 or control were stained with H&E. Tumor nodules were counted by a researcher blinded to the treatment groups on a light microscope.

### Statistical analysis

Non-parametric Mann–Whitney U-test was used to compare two groups, while one-way ANOVA followed by Dunnett’s or Tukey’s posthoc test was used to compare groups of three or more. Kaplan–Meier survival analysis was tested by log-rank test. Sample sizes for each experiment were based on a power calculation and/or previous experience of the mouse models. Analyses and data visualization were performed using GraphPad Prism (version 9.1.2) and Adobe Illustrator CS5.1 (version 15.1.0).

### Online supplemental material

The supplementary information shows gating strategy for γδ T cell isolation, and staining patterns of cell surface molecules (Fig. S1), signaling pathways not affected by PD-1 signaling (Fig. S2), pathway analysis of gene expression signatures in lung γδ T cells from tumor-bearing KB1P mice (Fig. S3), gating strategy and staining patterns of molecules identified by scRNAseq (Fig. S4), and additional data generated from the KB1P tumor model (Fig. S5). Table S1 lists differentially expressed genes between Clusters 1, 2.1, and 2.2 from Fig. 1 A. Table S2 lists differentially expressed genes between Clusters 1–7 from Fig. 5 A. Table S3 lists top pathways enriched in Clusters 1–7 from Fig. 5 A. Table S4 lists differently expressed genes between *Cd163l1*- and *5830411n06Rik*-expressing cells from Fig. 5 E. Table S5 lists differently expressed genes between CD27^−^ γδ T cells from WT and KB1P mice corresponding to Fig. 6 A.

## Supplementary Material

Table S1 lists differentially expressed genes between Clusters 1, 2.1, and 2.2 from Fig. 1 A.

Table S2 lists differentially expressed genes between Clusters 1–7 from Fig. 5 A.

Table S3 lists top pathways enriched in Clusters 1–7 from Fig. 5 A.

Table S4 lists differently expressed genes between *Cd163l1*- and *5830411n06Rik*-expressing cells from Fig. 5 E.

Table S5 lists differently expressed genes between CD27^−^ γδ T cells from WT and KB1P mice corresponding to Fig. 6 A.
